# Development of a Trauma-Informed, Culturally Sensitive Eating-Disorder-Specific Nutrition-Focused Physical Examination Tool: A Modified Delphi Study

**DOI:** 10.3390/nu17091449

**Published:** 2025-04-25

**Authors:** Donna Gallagher, Alainn Bailey, Laura Byham-Gray, Diane Rigassio-Radler, Jane Ziegler

**Affiliations:** Department of Clinical and Preventive Nutrition Sciences, School of Health Professions, Rutgers Health, Newark, NJ 07107, USA; eatpeacefully@gmail.com (D.G.); aab297@shp.rutgers.edu (A.B.); byhamgld@shp.rutgers.edu (L.B.-G.); rigassdl@shp.rutgers.edu (D.R.-R.)

**Keywords:** eating disorders, trauma-informed, culturally sensitive, nutrition assessment, nutrition-focused physical examination, body image, weight-inclusive, registered dietitian nutritionist, Delphi study

## Abstract

**Objective:** This study aimed to identify components of a trauma-informed, culturally sensitive eating-disorder-specific nutrition-focused physical examination (ED-NFPE) tool. **Method:** The modified Delphi methodology was used to develop a consensus on the clinical relevance of nine evidence-informed ED-specific nutrition examination domains and 46 components within those domains. Purposive sampling was used to recruit a panel of registered dietitian nutritionist (RDN) experts in the ED field. The panelists responded to survey statements on a five-point Likert scale. The panelists also provided qualitative feedback on domain and component additions, exclusions, modifications, and trauma-informed culturally sensitive examination practice techniques. **Results:** Twenty-two RDN expert panelists completed Round One of the study, and eighteen panelists completed Round Two (82% retention). Twenty-one were female. Fifteen panelists had ten or more years of experience in ED dietetics. Fifty percent held an advanced practice credential from the International Association of Eating Disorders Professionals (IAEDP). After the two survey rounds, the nine ED-NFPE domains and 46 of the 48 components achieved a consensus for clinical relevance. The panelists proposed two new examination components and provided qualitative feedback for trauma-informed culturally sensitive practice techniques in all nine domains. **Conclusions:** This modified Delphi study design was chosen to reach a consensus on developing an ED-NFPE tool, as there are few current evidence-based guidelines for nutrition examinations in ED care. An NFPE tool specifically designed to detect the nutrition-related findings of individuals with EDs would strengthen the overall nutrition assessment. RDNs at every level of care and with all degrees of experience could use an ED-NFPE to inform patient treatment goals.

## 1. Introduction 

Eating disorders (EDs) are complex biopsychosocial illnesses [1]. Individuals of every age, weight, shape, and size can develop an ED [2]. They often affect marginalized and vulnerable populations such as people of color, certain religious groups, people that are lesbian, gay, bisexual, transgender, queer, and others (LGBTQ+), and those with neurodivergence and physical disabilities [3,4,5,6,7]. Individuals with EDs may have co-occurring mental or medical illnesses and a history of trauma [8,9,10,11,12,13]. Nine percent, or 28.8 million Americans, will experience an ED in their lifetime [14]. The global lifetime ED prevalence is nearly eight percent [15].

**Figure 1:**
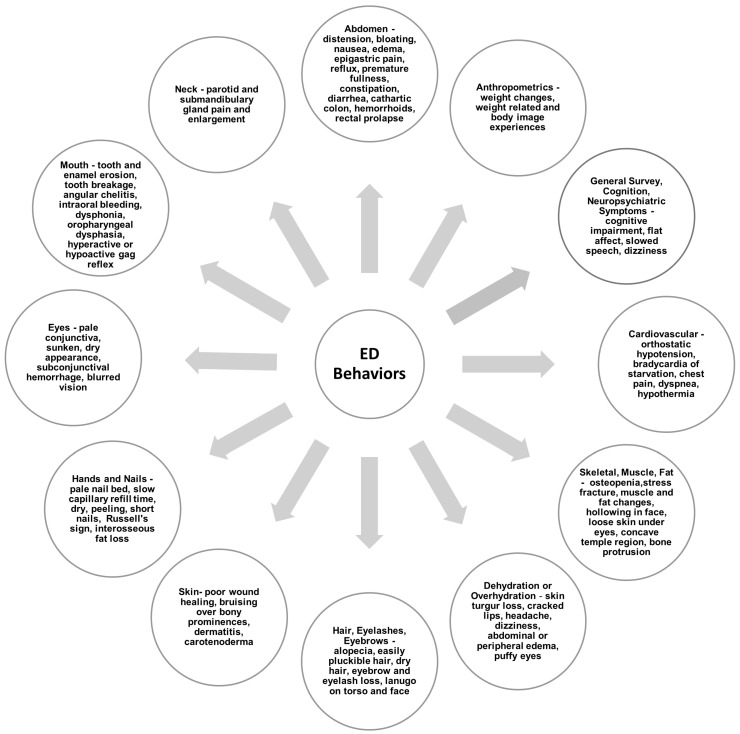
Nutrition-related signs and symptoms of eating disorder behaviors [13,16,17,18,19,20,21,22,23,24,25,26,29,30,31,32,33,34,35,36,37,38,39,40,41,42,43,44,45,46,47,48,49,50,51,52,53].

All ED subtypes are associated with nutrition-related medical complications, and al-though each individual with an ED may have a unique presentation, complications occur primarily due to ED behaviors such as restrictive eating, bingeing, purging, enema use, laxative, diuretic, and other substance abuse, overhydration, dehydration, excessive exercise, and others [2,16,17,18,19,20,21,22,23,24,25,26,27,28]. Complications of ED behaviors can be physical, psychological, and nutritional and include cardiorespiratory complications, bone, muscle, and fat changes, hydration and electrolyte imbalances, impaired cognition and psychosocial functioning, intraoral and extraoral issues, dermatologic abnormalities, parotid gland enlargement, gastrointestinal symptoms, and other complications affecting one or more organ systems (Figure 1) [13,16,17,18,19,20,21,22,23,24,25,26,29,30,31,32,33,34,35,36,37,38,39,40,41,42,43,44,45,46,47,48,49,50,51,52,53]. Awareness of unique ED-related complications, timely ED-specific nutritional, psychiatric, psychological, and medical assessments, and treatment are critical factors in ED recovery [54,55,56]. Most complications associated with EDs can be reversed through effective treatment, including the cessation of ED behaviors, proper nutrition rehabilitation, and weight restoration (if needed) [18,37,57].

In addition to a comprehensive assessment of an individual’s eating and exercise patterns, height and weight history, and family history of EDs or other psychiatric illnesses, the American Psychiatric Association (APA) recommends that multidisciplinary initial physical assessments of individuals with EDs be performed [58]. These include the evaluation of body temperature, heart rate, resting and orthostatic blood pressure, pulse, height, weight, body mass index (BMI), and physical signs of malnutrition and purging behaviors [58]. The APA also recommends a wide-ranging biochemical assessment, an electrocardiogram, and a complete review of body systems [58]. Lastly, the APA emphasizes that individuals with EDs should receive a culturally appropriate, person-centered treatment plan that includes psychological, psychiatric, nutritional, and medical experts [58].

Although national and international organizations recommend comprehensive assessments, there are few current ED-specific nutrition assessment tools, clinical guidelines, or standards for examining or assessing this population for nutrition-related complications [3,27,58,59,60,61,62,63,64,65,66,67,68,69,70]. Table 1 outlines the primary ED diagnoses, several ED behavior types, and co-occurring considerations for an ED nutrition assessment.

Registered dietitian nutritionists (RDNs) play a central role in ED care, as nutritional rehabilitation is one of the cornerstones of short- and long-term ED treatment [3,27]. The Academy of Nutrition and Dietetics (AND) standards of practice for RDNs include performing a comprehensive nutrition assessment, establishing a nutrition diagnosis(es), developing a nutrition intervention plan, monitoring and evaluating this plan, and conducting patient discharge or modifying the level of care provided [3,59,90,91].

The nutrition assessment domains of the nutrition care process include (1) client history, (2) anthropometric measurements, (3) biochemical, medical, and diagnostic tests, (4) food and nutrition history, (5) nutrition-focused physical examination (NFPE), and (6) ongoing reassessment(s), monitoring, and evaluation [3,42,59,63,64,70,90,92,93,94,95].

According to the APA, in individuals with EDs, guilt, embarrassment, and shame about ED behaviors and symptoms may prevent them from providing a complete and accurate food- and nutrition-related history [28]. Additionally, weight and body mass index (BMI) are not always diagnostic features of individuals with diagnoses other than anorexia nervosa, such as bulimia nervosa, binge eating disorder, “unspecified feeding or eating disorder”, and “other specified feeding or eating disorder”. These individuals may present in a “larger” body or with a BMI in a “healthy” range [28,63,66,96,97,98]. Lastly, individuals with EDs often present with normal biochemical and medical diagnostic test findings despite illness severity [3,27].

Because food and nutrition history, weight and BMI, and biochemical and medical diagnostic tests may not capture the physical signs and symptoms of malnutrition or the degree or severity of an ED, a systematic head-to-toe assessment tool (NFPE) is a crucial component of the nutrition assessment process [23,27,54,66,74,90,99].

The NFPE assessment tool evolved from the Subjective Global Assessment (SGA) tool and the American Society of Parenteral and Enteral Nutrition (ASPEN)/AND criteria for malnutrition [69,100]. The SGA is a validated nutrition assessment tool developed in the 1980s [100]. Based on patient history and physical examination findings, the SGA has been used by RDNs and other medical professionals globally to help determine the presence and severity of malnutrition [100].

In 2009, etiology-based diagnostic criteria for malnutrition were defined by the European Society for Clinical Nutrition and Metabolism (ESPEN) and, similarly, by a working group of the American Society of Parenteral and Enteral Nutrition (ASPEN) and the Academy of Nutrition and Dietetics (AND) [69,101]. The AND/ASPEN criteria were developed from the SGA and then expanded to include a minimum of two among the following items: insufficient energy intake, weight loss, loss of muscle mass and subcutaneous fat stores, fluid retention, and reduction in functional status [69,100]. Malnutrition was also defined in the context of acute illness or injury, chronic illness, or social or environmental circumstances [69].

The NFPE is utilized by RDNs in various clinical settings worldwide to assess micronutrient and macronutrient deficiencies, excesses, and imbalances and support a malnutrition diagnosis [28,64,92,93,102,103,104,105]. Muscle and fat stores, fluid retention (hydration status), functional status (grip strength), and other signs and symptoms of nutrition-related complications can be assessed through the NFPE [42,92,93,102,103,104]. The AND consistently updates the standards of practice and professional performance for RDNs (novice through to expert) and includes an NFPE to assess for malnutrition and overall nutrition status [3,59,70,92,102,103,104,106].

The examination techniques of inspection, palpation, percussion, and auscultation are used in the traditional NFPE to identify nutrition-related signs and symptoms arising from disease pathology [42,70,102,103,104,106]. The traditional NFPE domains include (1) general appearance, (2) vital signs, (3) skin, (4) nails, (5) head and hair, (6) eyes and nose, (7) mouth (intra and extraoral), (8) neck and chest, (9) abdomen, and (10) musculoskeletal system [92,93,102,104].

There are no evidence-based guidelines or standards for the NFPE of individuals with EDs [3,27,59,99]. The traditional NFPE lacks the specificity to thoroughly capture the unique nutritional complications commonly seen in individuals with EDs [93,99]. The conventional NFPE does not incorporate the principles of trauma-informed care, patient-centeredness, or culturally sensitive guidelines for its use in clinical practice, which are key considerations for providing the highest-quality healthcare (Table 2) [3,27,90,93,99,107,108,109,110].

According to the Substance Abuse and Mental Health Services Administration (SAMHSA), a trauma-informed approach in healthcare contributes to a positive treatment outcome. Trauma-informed care prioritizes the physical and emotional safety of survivors [109,110].

Although not all individuals with EDs have a trauma history, trauma safeguards must be instituted in ED assessments and treatment [115]. Individuals with a trauma history may have a heightened fear of threat, high emotional reactivity, and sensitivity to criticism [9]. A trauma history may also predict ED treatment withdrawal and poor recovery outcomes [9,115]. Alternatively, a patient’s perception of a safe and supportive treatment environment can help to remove obstacles to effective treatment [115].

Person-centered, culturally sensitive guidelines ensure that the treatment provider treats the patient as a unique person, not a diagnosis [107,108]. The treatment provider should exhibit awareness of their personal and professional biases, bear knowledge of various cultures, races, ethnicities, and religions, and understand the health disparities of marginalized groups [74,107].

A trauma-informed ED-specific nutrition-focused physical examination (ED-NFPE) tool, conducted with cultural sensitivity, could more thoroughly and adeptly identify nutrition-related complications in individuals with EDs and improve patients’ experiences and clinical outcomes [3,27,74,93,107,108,109,110]. The results of this ED-NFPE, in combination with other nutrition assessment findings from food and nutrition history, medical and psychosocial history, biochemical data, and medical and diagnostic tests and procedures, could help to inform goals for nutrition rehabilitation [3,27,74,90,99].

This study aimed to identify the domains and components of an ED-NFPE tool for RDNs to utilize at various levels of ED care to assess nutrition-related complications in individuals with EDs. The study also aimed to identify trauma-informed, culturally sensitive practice tools and techniques based on the principles of trauma-informed care in behavioral health science [110,116]. To meet these objectives, the study’s first aim was to conduct a literature review to identify evidence to inform the development of the ED-NFPE tool. The second aim was to develop a web-based survey and recruit ED RDN expert panelists [117,118,119,120]. The second aim used the modified Delphi methodology to establish a consensus among the expert panelists on the clinical relevance of the proposed ED-NFPE domains and components and to obtain feedback on a trauma-informed, culturally sensitive examination approach for inclusion in the ED-NFPE tool [121]. The research team employed the grounded theory research strategy to analyze the qualitative data and support the study objectives [122].

## 2. Materials and Methods

### 2.1. Study Design

#### 2.1.1. Delphi Approach Introduction

The Delphi and modified Delphi methods are widely used in mental health and ED research to develop clinical guidelines and improve mental health training, treatment, and cultural competence [60,65,123,124,125]. The modified Delphi method was chosen due to the complexities in the ED population and the need for expert opinions on ED care [121]. A modified Delphi approach, with iterative survey rounds and anonymous in-depth “discussion” and “communication” among expert panelists, will likely generate a strong consensus among the expert panel and reduce the impact of potential groupthink [121]. A literature review of the most commonly seen nutrition-related complications in EDs was conducted, and an analysis of the traditional NFPE domains and common nutrition-related complications in other clinical patient populations was performed. From the literature review, a pre-selected set of survey statements was developed. The modified method permitted a more focused study that could be conducted in six months with two to three rounds [121]. This type of study would also potentially maximize panelist participation and retention, as the time commitment was limited [121]. The researchers used the post-positivism research paradigm framework to obtain values-based, holistic, qualitative panelist feedback for trauma-informed, culturally sensitive examination techniques [126]. Three feedback rounds were determined a priori; however, only two were conducted to develop sufficient feedback and “communication” among panelists to achieve a consensus [121].

#### 2.1.2. Literature Review

An electronic literature review was conducted using the PubMed, CINAHL, and Scopus databases to identify primary research articles, review articles, and case studies on medical, dental, and nutrition assessment techniques and the current assessment methods used in ED care and to identify the common nutrition-related signs and symptoms resulting from the ED behaviors of all ED subtypes in children, adolescents, and adults.

The keywords and MeSH terms used in numerous groupings included “feeding and eating disorders” (MeSH), “eating disorders”, “anorexia nervosa”, “bulimia or bulimic or bulimia nervosa”, “avoidant restrictive food intake disorder or ARFID”, “atypical anorexia nervosa”, “pica”, “eating disorders not otherwise specified”, “physical assessment”, “nutrition assessment”, “nutrition-focused physical examination”, “skin conditions”, “skin signs”, “dermatologic signs”, “Russell’s sign”, “micronutrient deficiencies”, “macronutrient deficiencies”, “malnutrition”, “dehydration”, “overhydration”, “hydration”, “mouth symptoms”, “oral symptoms”, “vital signs”, “cognition”, “fat loss”, “muscle loss”, “hair”, “nails”, “eyes”, “neurological consequences”, “abdominal issues”, “parotid gland”, “cardiovascular”, “eating disorder treatment guidelines”, “gaps in eating disorders treatment”, and “eating disorder nutrition treatment guidelines”.

Several articles were hand-selected from the reference lists of primary research, review articles, and PubMed-suggested articles. Duplicate articles were eliminated. Published guidelines from international organizations such as the Academy of Nutrition and Dietetics, Academy for Eating Disorders, Australian and New Zealand Academy for Eating Disorders, American Psychological Association, American Psychiatric Association, and National Institute for Health and Care Excellence were retrieved from searches on Google Scholar, UpToDate, and reference lists from the chosen articles. Articles were also reviewed to clarify potential circumstances common in patients with eating disorders that should be addressed before and during the examination. These include patients in larger bodies, patients with a trauma history, racial, ethnic, and religious diversity, neurodiverse patients, LGBTQ+ patients, and patients with gastrointestinal diseases or other medical or mental health comorbidities.

The search was limited to articles written in English. The other inclusion criteria were years of publication from 1987 to 2025, adults, adolescents, and children, inpatient and outpatient settings, all genders, and all subtypes of eating disorders. Articles written before 2000 were included for historical value as relevant to the development of the NFPE.

The ED-specific nutrition-related complications identified through the literature search were cross-referenced with the traditional NFPE domains and common clinical findings in other clinical patient populations and organized into examination domains that were more ED-specific and could be conducted with the primary techniques of discussion and observation. The literature search identified nine ED-specific examination domains, including (1) anthropometrics, (2) general survey, cognition, and neuropsychiatric symptoms, (3) vital signs, (4) bone loss and injury, fat and muscle stores, (5) hydration status, (6) skin, hands, and nails, (7) hair, eyelashes, eyebrows, and eyes, (8) orofacial (intra and extraoral) and neck, and (9) abdomen (gastrointestinal) [92,93,102,104].

As an alternative to the traditional NFPE examination techniques of inspection, palpation, percussion, and auscultation, the examination techniques for the ED-NFPE tool included (1) discussion and (2) observation, (3) and measuring blood pressure, temperature, radial pulse, capillary refill rate, and grip strength (with explicit patient permission) [93,102]. Discussion and observation examination techniques (with explicit patient permission) were selected by the authors to accurately assess individuals for nutrition-related complications in most domains while adhering to the principles of trauma-informed care, patient-centered care, and cultural sensitivity (i.e., ensuring patient safety and maintaining appropriate boundaries) [3,27,74]. Measuring blood pressure, temperature, radial pulse, capillary refill rate, and grip strength requires physical contact to be performed and was, therefore, excluded from the observation and discussion technique categories and organized into a separate technique. The identified examination domains and common ED findings that informed all survey statements for an ED-NFPE tool are outlined in Table 3: Eating-Disorder-Specific Nutrition-Focused Physical Examination Tool.

The search also identified the six principles of trauma-informed care and culturally sensitive guidelines [58,74,107,108,109,110,116]. These principles and guidelines provided background to evaluate the panelists’ qualitative open-ended feedback. After validation through evidentiary support, the panelists’ responses will also inform the future development of trauma-informed, culturally sensitive instructional guidelines for ED-NFPE tools [3,4,5,6,8,12,13,23,50,63,72,74,75,76,77,78,79,80].

### 2.2. Survey Development

The literature search results informed the construction of the Qualtrics survey (Qualtrics, Provo, Utah), the “Eating Disorders Nutrition Assessment Tool” Survey (survey is available in Supplementary Information) [150]. The survey consisted of panelist consent, instructions, demographic and professional information, and a series of closed- and open-ended statements organized by examination domain. An introduction and description of each domain preceded the closed-ended statements. Each statement was designed so the panelists could rate their agreement with the clinical relevance of the domains and components on a 5-point Likert scale (strongly disagree, disagree, neither agree nor disagree, agree, or strongly agree) [123,151].

An open-ended statement requesting panelist feedback for domain and component addition, omission, or modification followed the set of closed-ended statements. Lastly, the survey included an open-ended statement for each domain, stating, “If you feel the (anthropometrics) domain is clinically relevant, please provide feedback on any particular practice tools or techniques you may currently employ or would recommend promoting a trauma-informed, culturally specific assessment. Leave blank if you prefer not to answer or have no additional feedback”.

### 2.3. Survey Distribution and Panelist Recruitment

The study was conducted from May through to August 2024. Purposive sampling was used to recruit a panel of RDN experts in ED practice. Several ED organizations were contacted to request their members’ email addresses for study participation or to post the study invitation on their professional discussion boards or professional interest group online forums. The organizations included in recruitment were the Academy for Eating Disorders (AED), the Academy of Nutrition and Dietetics (AND) Behavioral Health Nutrition Practice Group, Eating Disorder Registered Dietitians and Professionals (EDRD Pro), and the International Federation of Eating Disorder Dietitians (IFEDD) [117,118,119,120,152].

Organization leaders posted an invitation to participate in an initial screening survey on professional online forums or an invitation was directly emailed to nutrition professionals from these professional interest groups and ED organizations. The invitation included a brief introduction to the research study, research team, Delphi methodology, and study purpose. The screening survey included a brief set of questions to assess panelist eligibility.

If potential expert panelists met the criteria from the screening survey and chose to participate, they were sent a second personalized email including a link to the survey. Participants were informed of the time commitment of their participation. To maintain interest and reduce the risk of attrition, participants were offered the opportunity to participate in a raffle for a USD 50.00 Amazon e-gift card for their participation [153].

The inclusion criteria required that the expert panelists were RDNs with five or more years of experience in ED treatment or held an advanced practice credential through the International Association of Eating Disorders Professionals (IAEDP), with a minimum of 2500 supervised hours of work in the ED field [152]. Experts were not required to have advanced training in trauma-informed care. Although panelist group size can vary in Delphi studies, the study sought to recruit 20 or more panelists [123].

### 2.4. Ethics

The Rutgers University Institutional Review Board approved the study protocol and consent for panelists’ screening and participation (Pro2023002454). The authors followed the recommended Conducting and Reporting of Delphi Studies (CREDES) guidelines outlined by Jünger and colleagues and the Reporting guidelines for Delphi techniques in health sciences: A methodological review by Spranger et al. [154,155]. The panelists were informed that their demographic information and survey responses would be de-identified. Their anonymous responses would be included in future rounds for other panelists to review [156].

### 2.5. Survey Process

Round One included study background information, panelist consent, instructions, and demographic and professional information. The panelists were instructed to respond to statements about the clinical relevance of the nine ED-NFPE domains and 46 components within these domains by rating their agreement with the statements on a 5-point Likert scale [123,151]. The panelists were also given the option to provide feedback for domain and component addition, omission, modification, and practice tools or techniques they may currently employ or recommend to promote a trauma-informed, culturally specific ED-NFPE.

The panelists were required to complete and return their Round One survey via email within three weeks. After Round One data collection and analysis, the panelists were provided with a summary of their responses, mean group responses, and all other panelists’ de-identified responses to open-ended statements to permit the panelists to compare their responses to other panelists’ responses and revise them if desired. The panelists were given another week to review and revise their responses if desired. The round then closed, and a final data analysis was conducted to determine if consensus was achieved on any domains and components. Open-ended qualitative responses were reviewed for potential additions, omissions, modifications, and approaches. Survey items from Round One that the panelists added were verified through an additional literature review and added based on the results of a review of evidence. Feedback on a trauma-informed, culturally sensitive approach was analyzed for shared content. These comments will be evaluated for evidentiary support in future studies.

Round Two followed the same sequence as Round One. Items from Round One that achieved consensus were visible to the panelists in Round Two but were closed for further comments. Round Two contained statements that did not reach consensus in Round One and statements for the two additional components recommended by the panelists. As in Round One, the panelists were provided with a results summary and the opportunity to review and revise their responses if desired. Of the 48 final components, 46 reached a consensus after Round Two. There was no upward scoring trend for the additional two components in Round Two. It was, therefore, determined by the research team that a third round was not indicated. The results of Round Two determined a final consensus on domains, components, and approaches.

### 2.6. Data Analysis

IBM SPSS (IBM Corp. Released 2020. IBM SPSS Statistics for Windows, Version 27.0. Armonk, NY, USA: IBM Corp.) was used for data analysis [157]. Descriptive statistics (frequency and percentage) were used for panelist demographic information. The results for the clinical relevance of the ED-NFPE domains and components are reported as means and standard deviations (SDs). All research team members systematically read, hand-coded, and performed content analysis on the data from the qualitative responses to identify patterns in certain concepts or themes [122]. Based on previous Delphi studies on NFPE development, mental health, and ED practice, consensus was defined a priori as ≥75% of panelists answering “neither agree nor disagree”, “agree”, or “strongly agree” using the 5-point Likert scale, or as a rank of 4 ± 1 SD for each domain or component [65,151,158,159,160].

## 3. Results

### 3.1. Panelists

In total, 22 of the 44 panelists who participated in the eligibility screening survey met the eligibility criteria, consented to participate, and completed Round One (Table 4). Ninety-five percent identified as female. All panelists were 30 years old or older. Two-thirds had ten or more years of experience in ED dietetics. Fourteen worked thirty or more hours per week. Most panelists worked in an outpatient clinic or private practice. Three panelists were employed in a partial hospitalization program (PHP), intensive outpatient program (IOP), or “other” setting. All panelists were RDNs, with 50% holding an advanced practice credential from IAEDP. In the demographics portion of the survey, the panelists were not asked which country they lived in. They were, however, recruited from several international professional organizations, including IAEDP and IFEDD. Eighteen panelists completed Round Two (82% retention).

### 3.2. Round One

All nine domains (100%) and 44 of 46 components (95.6%) achieved consensus in Round One (Table 5). Two components, “BMI” and “measuring weight when weight restoration is not a treatment goal”, did not reach consensus.

Several panelists recommended two new components. These components, “capillary refill time” and “grip strength”, were reviewed for evidentiary support through a literature search and added to the Round Two survey in the “vital signs” and “bone, body fat, and muscle” domains, respectively.

From the open-ended statements, the panelists provided feedback for revisions, stating that the discussion and observation of components should be case-dependent and carefully considered and timed because of the topic sensitivity and to prevent the reinforcement of eating disorder cognitions. Additionally, the panelists suggested that other treatment providers should perform some measurements, observations, and discussions, especially if the RDN performs telehealth treatment. Several panelists felt that they needed additional training to assess and interpret some component findings.

The panelists were also asked to provide feedback, based on their extensive experience, on practice tools to promote a trauma-informed, culturally specific approach to examination components. Through hand-coding, frequently mentioned responses emerged (Table 6).

Each panelist was emailed a feedback form and could revise their ratings and responses by requesting a new survey link. Two panelists (9%) requested a new survey link and revised their responses.

### 3.3. Round Two

The statements that did not achieve a consensus on clinical relevance in Round One, body mass index (BMI) and “measuring weight when weight restoration is not a treatment goal” did not achieve consensus in Round Two (Table 7). The responses (N = 18) for these components remained low, with a frequency rating of ≤55.6%. The two newly recommended components, “assessing capillary refill rate” and “measuring grip strength”, achieved consensus, with frequency ratings of 88.9% and 100.0%, respectively. No additional additions, omissions, or revisions were suggested. Several new comments for a trauma-informed, culturally sensitive approach were offered, while various comments from Round One were re-stated in Round Two (Table 6).

As in Round One, the panelists were emailed personalized feedback forms and offered the opportunity to revise their responses. No panelists requested a new survey link for response revision.

At the end of Round Two, all nine assessment domains (100.0%) and 46 of the 48 (95.8%) final components achieved clinical relevance consensus with no ascendant response change in Round Two for the two remaining components, indicating no need for a third survey round. The research team evaluated the results and closed the study.

### 3.4. Domain 1—Anthropometrics

All panelists determined that Domain 1 was clinically relevant in an ED-NFPE (100%, n = 22). The panelists endorsed measuring height and growth trajectory using growth charts in children and adolescents and using BMI when plotting it against their growth curve, considering pubertal stage, family heritage, family eating patterns, and other medical and psychological considerations. Clarification was provided in Round Two that measurements in this domain and others could either be performed by the RDN or obtained by another clinician, shared with the RDN, and discussed with the patient in the nutrition assessment. The panelists proposed assessing medical conditions or medications that could affect weight and nutrient needs for all patients.

#### Panelist Feedback

In the open-ended statement where panelists could provide practice techniques promoting a trauma-informed, culturally specific assessment, multiple panelists recommended asking the patient for permission before weighing them, assessing their fears about being weighed, and discussing with the patient whether they (the patient) should be informed of their weight or if blind weighing (where the patient does not know the number) should be performed. When exact numbers may be triggering, the panelists recommended that the RDN use phrasing like “up, down, the same”, “moving in the right direction”, or “on target”. The panelists also suggested that the RDN convey to the patient that weight is only one component of the overall assessment and not the sole determinant of their health. Individuals, especially those with larger bodies and those who may have a history of weight-related emotional stress, weight bias, and weight shaming or bullying, should be permitted to provide consent for any discussion about weight, shape, size, weight history, and body image.

### 3.5. Domain 2—General Survey, Cognition, and Neuropsychiatric Symptoms

Domain 2 and its three components were unanimously rated as clinically relevant (100%, n = 22). The panelists recommended having access to other clinicians to provide additional details for the general survey, cognition, and neuropsychiatric symptom assessment. Some panelists suggested that other qualified professionals conduct this domain and that the results be shared with the RDN. The panelists indicated that in addition to asking the patient if they are taking medication regularly and as prescribed, they should ask if they have been prescribed any medication that they may not be taking as prescribed, as this might impact neuropsychiatric symptoms.

#### Panelist Feedback

The panelists stated that neuropsychiatric symptoms could lead to feelings of being misunderstood and potentially be harmful to some patients, particularly individuals who are neurodivergent. Therefore, this domain would need to be neuro-affirming. Additionally, the panelists suggested that the RDN should observe the patient’s ability to process and respond to questions or statements while asking the patient questions about these symptoms. To be accurately assessed, multiple panelists believed that all components in this domain should consider the involvement of environmental factors like trauma. By considering non-nutrition contributors, the effect of the patient’s nutritional status on cognitive and neuropsychiatric symptoms and changes may be more accurately determined.

### 3.6. Domain 3—Vital Signs

Vital signs achieved consensus (n = 22), with a 100% frequency rating. Four of the five components were unanimously rated as clinically relevant and achieved a consensus (100%, n = 22). Assessing capillary refill rate was added to the Round Two survey based on the panelist recommendations and additional literature review, and consensus (88.9%) was reached for clinical relevance. In addition to obtaining vital signs in a nutrition examination, several panelists recommended acquiring baseline, historical, or “normal” values from other treatment providers.

#### Panelist Feedback

The panelists suggested that the patient be informed of each component, how and why it will be performed, and that consent be provided before conducting any vital sign tests. The panelists stressed the importance of assessing the patient’s comfort level and giving reassurance about their autonomy and safety. According to the panelist feedback, blood pressure cuffs should be sized appropriately to accommodate individuals of all sizes and prevent potential traumatization. If preferred, the panelists proposed that the RDN may ask the patient to perform some of these tests independently or have them performed by a medical clinician and shared with the RDN. The panelists mentioned that RDNs who choose not to touch their patients can also ask a medical provider to measure blood pressure and heart rate.

### 3.7. Domain 4—Bone Loss and Injury, Body Fat, and Muscle Stores

This domain achieved consensus among all panelists (100%, n = 21). All five components achieved consensus, with frequency ratings between 77.3% and 100%. One panelist recommended an additional component, measuring grip strength. This component was added to Round Two and achieved consensus, with a frequency rating of 100% (n = 18).

The panelists recommended that the RDN observe and discuss any concerning changes during each session (e.g., muscle wasting, facial fat loss, etc.), not solely during the examination. The panelists affirmed that grip strength is an easily measured, non-invasive tool to assess malnutrition and can demonstrate muscle loss without emphasizing weight, shape, or body fat.

#### Panelist Feedback

The panelists recommended only conducting discussions with patients in this domain if relevant to ED recovery. According to the panelists, discussions should honor patients’ experience, autonomy, and safety, approaching each topic with consent and at a pace that meets the person where they can engage in the conversation. Body fat discussions can be re-traumatizing for some individuals with EDs. The clinical relevance of discussions on body fat and muscle should outweigh a patient’s distress. The panelists suggested that the RDN limits feedback on the patient’s body appearance but provides general education about the importance of adequate body stores.

### 3.8. Domain 5—Hydration Status

Hydration status achieved consensus (n = 21), with a 95.2% frequency rating. The discussion and observation of signs and symptoms of dehydration, overhydration, and abdominal and peripheral edema achieved unanimous consensus (n = 22), with a 100% frequency rating. There was some concern about the panelists’ inexperience in performing edema assessments and when providing telehealth; therefore, the panelists suggested that a medical provider perform the edema assessment. To assess objectivity, the panelists recommended screening for body dysmorphia before inquiring about edema.

#### Panelist Feedback

The panelists recommended screening for body dysmorphia before inquiring about swelling in the abdomen and ankles. Because “water loading” is common before a patient is weighed, the panelists suggested asking the patient to use the bathroom to urinate just before being weighed.

### 3.9. Domain 6—Skin, Hands, and Nails

This domain and its four components achieved unanimous consensus (100%, n = 22). Several panelists stated that RDNs could make clinically relevant observations in this domain, but may need additional training to detect signs and symptoms specific to EDs.

#### Panelist Feedback

Due to skin and nail quality variations among individuals of various races and ethnicities, the panelists suggested that the RDN should not make assumptions about a patient’s “normal” skin and nail quality. According to the panelists, the RDN should initiate a thoughtful discussion of nail and skin changes to ensure cultural competence. To be trauma-informed, the panelists stated that the RDN should be very clear and thoughtful before observing these components so that the patient feels safe and autonomous. The RDN should also discuss the context for the assessment and ask for explicit permission to observe the patient’s skin or hands before the evaluation.

### 3.10. Domain 7—Hair, Eyelashes, Eyebrows, and Eyes

This domain achieved consensus, with a 95.5% frequency rating. The six components achieved clinical relevance consensus, with frequency ratings between 86.4% and 100%. In addition to the RDN’s assessment, the panelists suggested that the patient’s perception of hair changes (including eyelashes and eyebrows) may also be clinically relevant. The panelists recommended that the RDN ask the patient if they take medications that might impact eye health.

#### Panelist Feedback

To assess transgender and gender non-conforming (TGNC) patients, the panelists recommended that the RDN be educated on the principles of gender-affirming care and the potential physical changes associated with gender transition. According to the panelist recommendations, for patients who have difficulty making eye contact (neurodiversity or trauma), the RDN should not require the patient to make eye contact during an eye assessment.

### 3.11. Domain 8—Intraoral, Extraoral, and Neck

The intraoral, extraoral, and neck domain and its six components achieved consensus, with frequency ratings between 86.4% and 100%. The panelists agreed that several critical micronutrient deficiencies are easily visible on the tongue and gingiva and suggested assessing chewing and swallowing issues.

The panelists were more comfortable assessing extraoral versus intraoral changes and more willing to discuss them without observing them. Several panelists stated that medical and dental providers should perform these assessments and share the results with the RDN. RDNs should, however, observe the parotid glands.

#### Panelist Feedback

The panelists stated that intraoral and extraoral areas are potentially personal and private areas of the body, especially in patients with a trauma history. Therefore, the RDN must be highly skilled and keenly aware of a possible adverse emotional response to this type of assessment. Skin color can impact mucous membrane and gingiva color; therefore, according to the panelists, the RDN should not make assumptions about a patient’s “normal” mucous membrane and gingiva color. The RDN should also ask the patient questions to ensure cultural competence.

### 3.12. Domain 9—Abdomen (Gastrointestinal)

This domain achieved unanimous consensus (100%, n = 22). The three components within this domain also achieved consensus (86.4–100%). Several panelists stated they would feel comfortable discussing abdominal and gastrointestinal symptoms, but would not observe the abdomen. They would refer the patient to a medical provider and request that they share the results.

#### Panelist Feedback

The panelists recommended including open-ended questions that permit discussions of race-specific or familial-specific gastrointestinal conditions or predispositions. They also suggested listening sensitively to patients’ concerns about their abdominal or gastrointestinal issues, even if the problem is not physiologically based. According to the panelists, RDNs should be aware that functional gastrointestinal issues are common in patients with EDs, and some patients with functional gastrointestinal disorders may have a history of sexual trauma.

## 4. Discussion

Individuals with EDs can experience unique, often life-threatening nutrition-related complications [16,20,21,39,57]. The early detection of these complications and a swift nutrition care plan implemented by an RDN are essential components of treatment [27]. Because the traditional NFPE may not thoroughly capture ED-specific nutrition-related complications, an ED-NFPE tool designed to detect these findings could improve the overall nutrition assessment and help to inform treatment [99].

The results of two similar studies indicate the need for disease-specific and population-specific NFPE tools for new and experienced RDNs in various specialties [159,160]. A modified Delphi study was conducted in 2022 by Pike et al. to establish a consensus on the components of the traditional NFPE that would be clinically relevant for detecting the nutrition-related conditions seen explicitly in athletes [159]. In 2023, a modified Delphi study was conducted by Bathgate et al. to examine the need for a modified pediatric NFPE tool specific for infants and children with bronchopulmonary dysplasia (BPD) [160].

In this study, 22 RDN panelists in Round One and 18 in Round Two found the nine ED-NFPE examination domains and 46 of 48 domain components to be clinically relevant for incorporation into an ED-NFPE. Primary ED-NFPE techniques of discussion and observation (with explicit patient permission) were proposed in the place of inspection, palpation, percussion, and auscultation, the traditional NFPE techniques. By conducting a less “invasive” examination, the RDN can assess nutrition-related complications adeptly while ensuring patient autonomy and safety and respecting religious and cultural rules and preferences. The results indicated that the expert panelists predominantly supported these examination techniques.

A unique feature of this study was the qualitative panelist feedback provided for trauma-informed, culturally sensitive practice techniques. The RDN panelists recognized potential trauma and its impact on an individual’s treatment experience. They provided qualitative responses to minimize the risk of inadvertent re-traumatization and optimize treatment adherence and outcomes [9,110,116].

The panelists also provided numerous suggestions for providing a culturally sensitive NFPE, including maintaining a weight-inclusive treatment approach, assessing a patient’s comfort level around weight and body image, and conveying that weight is not the sole determinant of health (state vs. weight) [161].

Although the NFPE is an assessment tool used by RDNs in various clinical settings and performed on numerous patient populations, a common theme in the panelists’ comments was a lack of comfort in performing some examination techniques in several domains. Some panelists stated they would feel comfortable discussing but not observing (i.e., abdominal and intraoral) specific symptoms and observing but not discussing (body fat and muscle) others. They would prefer that a medical provider perform some examination components and share the results with the RDN. The panelists also felt that they needed more training to perform some examination components. Lastly, the panelists who performed telehealth expressed limitations regarding what examination components they could perform.

### 4.1. Strengths and Limitations

Although the selection of domains and components for the ED-NFPE was based on an extensive literature search, a limitation of this study is that the researchers may have unintentionally introduced bias in statement wording and the choice of ED-specific domains and examination techniques. RDN panelists unfamiliar or uncomfortable with the domains and components may have introduced bias into their survey responses, and their answers may have been influenced by their personal or professional bias [125]. Although all panelists had wide-ranging experience in ED care, it was unclear if they had extensive experience in trauma-informed care or exhibited cultural, racial, or other forms of diversity, possibly introducing bias into their responses. Detailed narratives preceded survey statements. However, the panelists may not have completely understood the statements, and an opportunity for statement clarification was only made available in the following round (Round Two) [65].

Another limitation was that consensus cutoffs included neutral ratings. “Neither agree nor disagree” responses may not have indicated that the panelists supported the statement. Lastly, while panelist group size varies between 10 and 100 in most published Delphi studies, a limitation of this study is the panelist group size of 22 [123].

A significant strength of this study is that consensus was achieved in all nine domains and 46 of the final 48 components. Another strength and core objective of the study is the extensive qualitative feedback that the panelists provided in all domains. The retention rate was high, with 22 panelists in Round One and 18 panelists in Round Two (81.8% retention).

### 4.2. Implications of Findings

A thorough trauma-informed, culturally sensitive ED-NFPE tool will close the gap in ED patient care by enabling RDNs to skillfully conduct detailed, comprehensive nutrition assessments of individuals in this population. An ED-NFPE could be utilized at the onset and throughout treatment to evaluate changes in nutritional status during the re-nourishment and recovery process. RDNs could use this tool at the individual outpatient level of care through to the acute inpatient treatment level.

RDNs in various clinical and non-clinical settings and those with limited ED expertise could use an ED-NFPE tool as a resource to help identify potential ED-related nutritional findings, provide appropriate treatment referrals, and improve patient care.

Several factors, including a lack of experience or knowledge of the NFPE, time constraints, and a lack of confidence, autonomy, and training, may limit RDNs’ use of the NFPE [162,163]. Formal NFPE training for general practice RDNs, RDNs in ED care, and students enrolled in accredited dietetics programs could provide ample knowledge and hands-on experience to remove potential barriers to conducting comprehensive disease-specific or population-specific nutrition examinations, such as an ED-NFPE [164].

## 5. Conclusions

In-depth ED-specific assessments and multidisciplinary treatment are vital to reduce or eliminate ED behaviors and minimize, eliminate, or reverse potential nutrition-based medical complications [3]. The RDN is a crucial treatment team member at every level of care [3,27]. To provide optimal ED nutrition care, evidence-based standards and guidelines must be developed for RDNs. The results of this study show that a trauma-informed, culturally competent ED-NFPE tool can not only improve the nutrition assessment of individuals with EDs, but can potentially improve the overall patient experience.

Future studies can build on this study’s results to develop and validate ED-NFPE tool components and evidence-based guidelines for their use, explore the tool’s impact on patient outcomes, and obtain patient feedback on the examination experience. The further development and validation of an ED-NFPE tool and professional NFPE training for RDNs will help to direct nutrition interventions and improve patient outcomes.

## Figures and Tables

**Table 1:** Eating disorder diagnoses, behavior types, and co-occurring considerations.

Eating Disorder Diagnoses [28]	Eating Disorder Behavior Types [27,28,45,71,72,73]	Co-occurring Considerations [2,4,12,13,28,74,75,76,77,78,79,80,81,82]
Anorexia nervosa restricting type	Restrictive eating [28]	Anxiety disorders [12]
Anorexia nervosa binge/purge type	Bingeing [28]	Bipolar disorder [12]
Avoidant/restrictive food intake disorder	Purging [28]	Borderline personality disorder [12]
Binge eating disorder	Laxative/enema abuse [27]	Major depressive disorder [12]
Bulimia nervosa	Diuretic abuse [27]	Obsessive-compulsive disorder [12]
Other specified feeding or eating disorder	Overhydration	Autism spectrum disorder [5,7]
Pica	Dehydration [20]	Attention-deficit disorder [5]
Rumination disorder	Chewing and spitting [27]	Intellectual developmental disorder [5]
Unspecified feeding or eating disorder	Ipecac abuse [20]	Substance use disorders [12]
	Regurgitating and re-chewing food [28]	Trauma history [10]
	Over exercise [27]	Associated medical conditions [13]
	Night eating [83]	Socioeconomic status/food insecurity [84]
	Insulin manipulation (type 1 diabetes) [85]	Age [66,77]
	Eating non-food items [28]	Biological sex [2]
	Supplement misuse [27]	Gender identity [4]
	Alcohol or drug abuse [86]	Sexual orientation [4,80]
		Body weight and size [87,88]
		Race [74]
		Ethnicity [74]
		Religion [74]
		Physical disability [74]
		Athlete [75]
		Extreme concern over weight, shape [89]

**Table 2:** Principles of trauma-informed care, patient-centeredness, cultural sensitivity.

Trauma-Informed Care [109,110]	Patient Centeredness [107,108]	Cultural Sensitivity [74,107]	Unique Considerations for TIC and Cultural Sensitivity [4,6,64,74,78,79,87,88,111,112,113]
Create a physically and emotionally safe environment	Regard the patient as a person, not a diagnosis	Understand the patient’s values, beliefs, and treatment preferences	LGBTQ+ [4,64,111,113,114]—medical trauma is common; stigma in health care is often experienced; gender-affirming communication is essential.
Establish trust, clarity, and appropriate boundaries	Share responsibility and power with the patient	Build relationship trust and boundaries	Individuals in higher-weight bodies [6,87,88]—independent of weight, body image concerns can impact self-esteem and lead to ED behaviors; weight stigma can reduce quality of care; may have severe symptoms despite body weight or size.
Assure patients they have choice and control, and emphasize patient empowerment	Build an appropriate provider–patient relationship	Bear in mind one’s own biases and beliefs	Religious groups [74,78,79,112]—religious rules may play a role in food choices and fasting, causing short-term deficiencies unrelated to ED behaviors; some religions have guidelines around modesty, upholding no physical contact, while thoroughly covering the head and/or body with headwear and specific clothing. These guidelines necessitate the RDN’s exhibition of respect.
Build a collaborative relationship while sharing power	Express unconditional positive regard and respect toward the patient	Possess knowledge and understanding of various cultures and minority groups	Racial and ethnic differences [74,79]—there are potential differences in the presentation, signs, and symptoms associated with EDs in BIPOC; dark-skinned individuals are also more prone to skin conditions like xerosis and acanthosis nigricans and vitamin D deficiency than light-skinned individuals.
Encourage the support of others who have a history of trauma			
Provide services that are inclusive of individuals with various backgrounds while avoiding stereotypes and biases			

TIC—trauma-informed care; BIPOC—black, indigenous, and people of color.

**Table 3:** Eating-Disorder-Specific Nutrition-Focused Physical Examination Tool.

Examination Domain and Components	Examination Technique	Normal Findings	Common ED-Specific Examination Findings	Possible Nutritional Causes	Comments/ Non-Nutritional Causes
Anthropometrics	Discussion of weight, weight history, and weight-related experiences Measure height and weight (with explicit patient/guardian permission) when weight restoration is a treatment goal, and measure against previous growth charts (if child or adolescent) [63]	Normal weight for height, weight, height, and stature for age and developmental stage, following previous growth curve trajectory (if child or adolescent) [93,99]	Weight loss, weight gain, variation from prior growth curve trajectory (if child or adolescent), and history of weight bullying and/or weight stigma [18,63,127]	Malnutrition, excessive exercise, binge eating, weight-related eating disorder antecedents, current weight-related challenges, and non-nutrition-related weight-related circumstances [23,44,63]	Medications and non-nutrition related illnesses [27]
General Survey Neuropsychiatric, Cognition, and Mood	Discussion and observation of engagement and alertness	Engagement and alertness [93,99]	Lack of engagement, weakness, sleep disturbances, and fatigue [44,50,63]	Malnutrition, dehydration, excessive exercise, and night eating syndrome [18,19,20,44,63,71,81]	Neurodiversity, autism spectrum disorders, ADHD, or other learning disabilities, and medication type and dose [5,27]
	Discussion and observation of cognition, mood, and speech	Appropriate answers to questions, age-appropriate behavior, and cognition [93,99]	Depressed mood, feels cold, memory loss, cognitive impairment, flat affect, anxiety, dizziness, slowed speech, tingling of hands and/or feet, skeletal muscle cramps or paralysis, and nerve palsy [18,44,45,50,63,81]	Thiamine, pyridoxine, vitamin B12 deficiencies, macronutrient deficiencies, calorie deficiency, dehydration, medication thyroid medication misuse, hypothyroidism, and excessive exercise [18,19,20,44,63,71,81]	Neurodiversity, autism spectrum disorders, ADHD, or other learning disabilities, and medication type and dose [5,27]
Vital signs and temperature	Discussion and obtain radial pulse (heart rate), blood pressure (BP) (supine to sitting or sitting to standing), capillary refill rate, and temperature (with explicit patient/guardian permission)	Pulse: 60–100 pulses/min BP: <130 mmHg/85 mmHg Capillary refill time: <2 s Temperature: 96.4–99.1 °F [93,99]	Bradycardia (<60 pulses/min in adults, <50 pulses/min in adolescents and children), tachycardia > 100 pulses/min in adults, arrhythmia, orthostatic hypotension (decrease in systolic BP of 25 mmHg from supine to sitting or sitting to standing in adults, decrease in systolic BP of >20 mmHg, and a decrease in diastolic BP of >10 mm/Hg (for children and adolescents), hypertension, hypothermia, cool extremities, lightheadedness, chest pain, fatigue, hot flashes, dyspnea, and capillary refill time ≥ 2 s [20,33,63,128,129,130]	Malnutrition, weight loss, dehydration, purging, excessive exercise, laxative or diuretic abuse, thyroid medication misuse, electrolyte imbalances (hypokalemia, hypomagnesemia, hypophosphatemia), refeeding syndrome, and hypothyroidism (euthyroid sick syndrome) [20,21,44,63,129,131,132]	Antipsychotic, antidepressant, and mood stabilizer medications [128]
Bone Loss and Injury Fat Stores Muscle Stores	Discussion of potential bone loss, fracture, or injury	No bone loss or age-related bone loss and no fracture or bone-related injury	Osteopenia, osteoporosis, fracture, and over-exercise-associated stress fracture or injury [49,133]	Micronutrient and macronutrient deficiency, low estrogen levels, hypothyroidism, and over-exercise [134]	Age-related osteoporosis or athletic injury [135,136]
	Discussion and observation (with explicit patient/guardian permission) of the orbital region	Minimal fat wasting and slight bulge in the orbital fat pad [93,99]	Reduction in fat stores in all regions, including the face, hollowing, dark color, loose skin under eyes, and delayed pubertal development [41,63]	Macronutrient and calorie deficiencies and excessive exercise [23,44,63]	Hormone replacement therapy in gender-affirming treatment can alter body composition [137]
	Discussion and observation (with explicit patient/guardian permission) of interosseous hand muscles, temple region, and grip strength measurement	Firm, well-defined muscles of normal size and shape bilaterally [93,99]	Changes in body composition, decreased muscle mass, muscle weakness, reduced grip strength, muscle pain, hollow, flattened muscle, concave temple region, cramping, rhabdomyolysis, and delayed pubertal development [20,41,63,138,139,140]	Protein and calorie malnutrition, excessive exercise, dehydration, and laxative abuse [23,44,63]	Hormone replacement therapy in gender-affirming treatment can alter body composition [137]
Hydration Status	Discussion and observation (with explicit patient/guardian permission) of hands, feet, ankles, abdomen, and eyelids	Normal mucus membranes and no edema [93,99]	Dehydration—sunken eyes, dark area under eyes, loss of skin turgor, dry, cracked lips, headache, dizziness, concentrated urine, and overhydration—puffy eyes, edema in abdominal area, peripheral edema, and frequent urination [20,21,38,128,141]	Lack of appropriate fluid intake, excessive fluid intake (water loading), excessive caffeine intake, protein deficiency, thiamin deficiency, purging, laxative or diuretic abuse, ipecac abuse, insulin misuse, excessive exercise, pseudo-Bartter’s syndrome, rebound or refeeding edema, and refeeding syndrome [20,21,44,52,128]	Include a detailed discussion of fluid, caffeine, diuretic, and laxative intake in food/nutrition history to determine if the patient uses fluid and caffeine to mask hunger
Skin Hands and Nails Hair, Eyelashes, and Eyebrows Eyes	Discussion and observation (with explicit patient/guardian permission) of skin	Uniform color, texture, moisture, and temperature [93,99,102]	Carotenoderma on palms of hands, poor wound healing, pallor, xerosis, acanthosis nigricans, hirsutism, dermatitis, acne, loss of turgor, cool temperature, scars on backs of hands from purging, general bruising, bruising over bony prominences, striae distensae, cyanosis, signs of self-harm (burns, cuts—especially on arms, legs, and abdomen), and dermatitis artefacta [20,29,63,142]	Iron, folate, vitamin B12 deficiency, zinc deficiency, macronutrient deficiencies or excesses, poor fluid status, fat deficiency, vitamin A deficiency, excessive intake of beta-carotene-containing foods, essential fatty acid deficiency, calorie deficiency, purging, peripheral vasoconstriction, excessive exercise, and binge eating [29,44,63,102]	Increased facial hair from hormone replacement therapy (transgender patients) [137] and self-harm
	Discussion and observation (with explicit patient/guardian permission) of hands and nails	Smooth, standard color and shape, and less than two seconds capillary refill time (CRT) [93,99,102]	Ridges, koilonychia, dry, peeling, or short nails, pale nail bed, bleeding cuticles, interosseous muscle loss, poor circulation, Russell’s sign, and slow CRT [29,63,102]	Iron, protein, zinc, folate, magnesium, selenium deficiency, macronutrient, micronutrient, and calorie deficiency, dehydration, and purging hypovolemia [29,102]	Nail and cuticle biting
	Discussion and observation (with explicit patient/guardian permission) of hair, eyes, eyelashes, eyebrows, and fat pads below the eyes	Uniform color, texture, and amount of hair, eyelashes, and eyebrows [93,99]	Alopecia, easily pluckable, brittle, dry hair, loss of eyebrows and eyelashes, and lanugo on face and torso [29,63,102]	Macronutrient, iron, zinc, essential fatty acid deficiency, hypothyroidism, severe malnutrition, and weight loss [102]	Trichotillomania, antidepressants [102], and hormone replacement therapy (transgender patients) [137]
		Clear conjunctivae, moist, and pink membranes [93,99]	Pale conjunctivae, sunken, dry appearance, and subconjunctival hemorrhage [20,63,143]	Dehydration, vitamin A deficiency, iron deficiency, fat loss, and purging [143]	
Mouth Neck	Discussion and observation (with explicit patient/guardian permission) of teeth, lips, gums, mucosa, tongue, breath, and voice	A normal amount of healthy teeth, pink, smooth lips with no sores, pink gums, red, moist tongue with papillae, and a rough appearance [93,99,102,104]	Tooth erosion, enamel erosion, tooth sensitivity, tooth breakage, missing teeth, dental caries, angular cheilitis, red, cracked lips, dark red (magenta) tongue, excessive or minimal saliva production, intra and extraoral mouth sores, palatal scratches or ulcer, gingival recession, swollen bleeding gums, redness in back of mouth/throat, oral bleeding, oropharyngeal dysphasia, halitosis, hyperactive or hypoactive gag reflex, and hoarse voice [18,20,22,30,32,39,43,47,63,102,104,143,144]	Macronutrient deficiency, bingeing, purging, biting, chewing on hard foods (ice and hard candy), using hard implements to purge (i.e., toothbrush), eating non-food items, chewing and spitting, consuming large amounts of food in a short period of time (binge eating), regurgitation of stomach acid, iron, riboflavin, niacin, pyridoxine, vitamin B12, folate, deficiency, and ketoacidosis [20,47,102,104,145]	
	Discussion and observation (with explicit patient/guardian permission) of the neck	Fatty, triangular shape, and unilobular [93,146]	Parotid and submandibular gland pain and enlargement [63,147]	Bingeing and purging [63,147]	
Abdomen	Discussion and observation (with explicit patient/guardian permission) of the abdomen	Flat, round, or scaphoid appearance, normal bowel sounds, and minimal self-reported discomfort [93,99]	Distension, bloating, nausea, fluid accumulation, flatulence, epigastric discomfort, extreme scaphoid abdomen, constipation, blood in stool, rectal fissure, reflux, hard abdomen, early satiety, gastric dilatation, constipation, diarrhea, cathartic colon, hemorrhoids, and rectal prolapse [18,63,81,144,148]	Gastroparesis, irritable bowel syndrome, celiac disease, Crohn’s disease, muscle loss and fat loss from malnutrition, excessive exercise, superior mesenteric artery syndrome, laxative abuse, pelvic floor dysfunction, binge eating, and straining from constipation [11,39,44,53,63,81,143,149]	Antidepressant medications, anti-anxiety medications, antipsychotic medications, and mood stabilizers

**Table 4:** Demographics and professional characteristics of eating disorder dietitian expert panelists.

Variable (N = 22)	*n*	%
**Gender**		
Female	21	95.5
**Age**		
30–39	7	31.8
40–49	5	22.7
50+	9	40.9
**Years Employed in Eating Disorders Dietetics**		
5–9 years	7	31.8
10–14 years	3	13.6
15 years or more	12	54.5
**Professional Practice Status**		
Full-time (≥30 h/week)	14	63.6
Part-time (≤29 h/week)	6	27.3
Other	2	9.1
**Eating Disorders Treatment Setting Where Employed**		
Outpatient Clinic or Private Practice	19	86.4
Other	3	13.6
**Nutrition Profession**		
Registered Dietitian Nutritionist	22	100.0
**International Association of Eating Disorders Professionals (IAEDP) Accreditation**		
Yes	11	50.0
No	11	50.0
**IAEDP Advanced Practice Credential**		
Certified Eating Disorder Specialist (CEDS)	6	27.3
Certified Eating Disorder Specialist-Consultant (CEDS-C)	5	22.7

**Table 5:** Round One domains’ and components’ clinical relevance, consensus determination.

Domains and Components of Examination	Clinical Relevance Group Mean (N = 22)	Standard Deviation	Frequency (%) Rating 3 to 5	Consensus
**Domain 1 Anthropometrics**	4.55	0.51	100.0	yes
**Components A1–A6 Anthropometrics**				
A1 BMI	2.59	1.30	59.0	no
A2 Measuring height and growth trajectory	4.95	0.21	100.0	yes
A3 Measuring weight when weight restoration is a goal	4.68	0.57	100.0	yes
A4 Discussing weight changes, body image, and weight experiences when weight restoration is a goal	4.68	0.48	100.0	yes
A5 Measuring weight when weight restoration is not a goal	2.00	0.93	22.7	no
A6 Discussing weight changes, body image, and weight experiences when weight restoration is not a goal	3.86	1.39	77.3	yes
**Domain 2 General Survey, Cognition, and Neuropsychiatric Symptoms**	4.95	0.21	100.0	yes
**Components G1–G3 General Survey, Cognition, and Neuropsychiatric Symptoms**				
G1 General Survey Assessment	4.95	0.21	100.0	yes
G2 Cognitive Assessment	4.82	0.40	100.0	yes
G3 Neuropsychiatric Symptoms Assessment	4.86	0.35	100.0	yes
**Domain 3 Vital Signs**	4.86	0.35	100.0	yes
**Components V1–V5 Vital Signs**				
V1 Discussing vital signs and symptoms	4.91	0.29	100.0	yes
V2 Measuring blood pressure	4.60	0.73	100.0	yes
V3 Measuring orthostatic blood pressure	4.82	0.50	100.0	yes
V4 Measuring heart rate using radial pulse	4.73	0.55	100.0	yes
V5 Measuring temperature	4.09	1.15	86.3	yes
**Domain 4 Bone Loss and Injury, Body Fat, and Muscle Stores**	4.62 (n = 21)	0.59	100.0 (n = 21)	yes
**Components B1–B5 Bone Loss and Injury, Body Fat, and Muscle Stores**				
B1 Discussing bone health	4.77	0.43	100.0	yes
B2 Discussing body fat changes	3.64	1.22	77.3	yes
B3 Observing body fat changes	3.77	1.11	86.4	yes
B4 Discussing body muscle changes	4.05	0.95	90.9	yes
B5 Observing body muscle changes	4.14	0.89	90.9	yes
**Domain 5 Hydration Status**	4.81 (n = 21)	0.40	95.2 (n = 21)	yes
**Components H1–H8 Hydration Status**				
H1 Discussing dehydration signs and symptoms	4.68	0.65	100.0	yes
H2 Observing dehydration signs and symptoms	4.86	0.35	100.0	yes
H3 Discussing overhydration signs and symptoms	4.59	0.67	100.0	yes
H4 Observing overhydration signs and symptoms	4.73	0.46	100.0	yes
H5 Discussing abdominal edema signs and symptoms	4.18	0.91	100.0	yes
H6 Observing abdominal edema signs and symptoms	4.41	0.80	100.0	yes
H7 Discussing peripheral edema signs and symptoms	4.36	0.79	100.0	yes
H7 Observing peripheral edema signs and symptoms	4.55	0.67	100.0	yes
**Domain 6 Skin, Hands, and Nails**	4.41	0.67	100.0	yes
**Components S1–S4 Skin, Hands, and Nails**				
S1 Discussing skin and hands	4.27	0.70	100.0	yes
S2 Observing skin and hands	4.36	0.79	100.0	yes
S3 Discussing nails	4.18	0.73	100.0	yes
S4 Observing nails	4.32	0.78	100.0	yes
**Domain 7 Hair, Eyelashes, Eyebrows, and Eyes**	4.23	0.87	95.5	yes
**Components E1–E6 Hair, Eyelashes, Eyebrows, and Eyes**				
E1 Discussing hair	4.27	0.63	100.0	yes
E2 Observing hair	4.27	0.83	95.2	yes
E3 Discussing eyebrows and eyelashes	3.59	1.01	86.4	yes
E4 Observing eyebrows and eyelashes	3.82	1.14	86.4	yes
E5 Discussing eyes	3.95	0.90	100.0	yes
E6 Observing eyes	4.18	0.96	95.5	yes
**Domain 8 Intraoral, Extraoral, and Neck**	4.14	1.08	86.4	yes
**Components O1–O6 Intraoral, Extraoral, and Neck**				
O1 Discussing intraoral	4.05	0.90	95.5	yes
O2 Observing intraoral	3.91	1.11	86.4	yes
O3 Discussing extraoral	3.95	1.00	90.9	yes
O4 Observing extraoral	4.00	1.20	81.8	yes
O5 Discussing neck	3.95	0.79	100.0	yes
O6 Observing neck	4.14	0.89	95.5	yes
**Domain 9 Abdomen (Gastrointestinal)**	4.86	0.35	100.0	yes
**Components G1–G3 Abdomen (Gastrointestinal)**				
G1 Discussing gastrointestinal signs and symptoms	4.82	0.40	100.0	yes
G2 Discussing abdomen	4.50	0.67	100.0	yes
G3 Observing abdomen	4.09	1.11	86.4	yes

BMI—body mass index.

**Table 6:** Rounds One and Two feedback for trauma-informed, culturally sensitive techniques.

Domain	Feedback *
Anthropometrics	The RDN should ask the patient for permission before weighing them, assess their fears about being weighed, and discuss with the patient whether they (the patient) should be informed of their weight or if a blind weight (where the patient does not know the number) should be performed. When exact numbers may be triggering, the RDN should use phrasing like “up, down, the same”, “moving in the right direction”, or “on target”. The RDN should convey to the patient that weight is only one component of the overall assessment and not the sole determinant of their health. The RDN should adopt a weight-inclusive approach when discussing concerns about body image. Individuals, especially those with larger bodies and those who may have a history of weight-related emotional stress, weight bias, and weight shaming or bullying, should be permitted to provide consent for any discussion about weight, shape, size, weight history, and body image.
General Survey, Cognition, and Neuropsychiatric Symptoms	Neuropsychiatric symptoms could lead to feelings of being misunderstood and potentially be harmful to some patients, particularly individuals who are neurodivergent. Therefore, this domain would need to be neuro-affirming. While asking the patient questions about these symptoms, the RDN should observe the patient’s ability to process and respond to questions or statements. The RDN should discuss social connection, the patient’s willingness and ability to engage in everyday social activities (after-school activities, friend gatherings, church, family gatherings, etc.), and the frequency of “meltdown/shutdown” experiences in clients with autism spectrum disorders. All components in this domain should consider the involvement of environmental factors like trauma. By considering non-nutrition contributors, the effect of the patient’s nutritional status on cognitive and neuropsychiatric symptoms and changes may be more accurately determined.
Vital Signs	The RDN should inform the patient of each component and explain how and why it will be performed. The patient should provide consent before any vital sign tests are conducted. The RDN should assess the patient’s comfort level and reassure them of their autonomy and safety. Blood pressure cuffs should be sized appropriately to accommodate individuals of all sizes and prevent potential traumatization. The RDN may ask the patient to perform some of these tests independently if the patient prefers to do so. RDNs who prefer not to perform these components or choose not to touch their patients (radial pulse and blood pressure) can ask the patient to have their medical provider perform them and share the results with the RDN.
Bone Loss and Injury, Body Fat, and Muscle Stores	The RDN should only conduct discussions with patients in this domain if relevant to ED recovery. Discussions should honor the patient’s experience, autonomy, and safety, and each topic should be approached with consent and at a pace that meets the person where they can engage in the conversation. Body fat discussions can be re-traumatizing for some individuals with EDs. The clinical relevance of discussions on body fat and muscle should outweigh the patient’s distress. Assessment results should be considered in the context of family frame size and body type. When weight restoration and nutrition rehabilitation are necessary, body fat and muscle changes should be discussed before changes occur to help the patient understand what to expect during the nutritional rehabilitation process. Because the topics of body fat and body muscle are sensitive topics for individuals with EDs, discussions should be case-dependent and primarily used when the loss of body muscle and fat is a concern. Discussion may be more relevant than observation (with specific exceptions such as orbital fat stores) The RDN could provide body fat and body muscle information as a component of exposure therapy, but must be mindful not to reinforce ED cognitions. Due to anti-fat bias, conversations regarding fat stores may be more sensitive than discussions on muscle or bone health. The RDN should limit feedback on the patient’s body appearance but provide general education about the importance of adequate body stores.
Hydration Status	The RDN should screen for body dysmorphia before inquiring about swelling in the abdomen and ankles. Because “water loading” is common before a patient is weighed, the RDN should ask the patient to use the restroom before the weigh-in.
Skin, Hands, and Nails	Due to skin and nail quality variations among individuals of various races and ethnicities, the RDN should not make assumptions about a patient’s “normal” skin and nail quality. The RDN should initiate a thoughtful discussion of nail and skin changes to assure cultural competence. The RDN should be clear and thoughtful before observing components in this domain so that the patient feels safe and autonomous. The RDN should also discuss the context for the assessment and ask for explicit permission to observe the patient’s skin or hands before the evaluation.
Hair, Eyelashes, Eyebrows, and Eyes	To assess TGNC patients, the RDN should be educated on the principles of gender-affirming care and the potential physical changes associated with gender transition. For patients who have difficulty making eye contact (neurodiversity or trauma), the RDN should not require the patient to make eye contact during an eye assessment.
Intraoral, Extraoral, and Neck	The RDN must be highly skilled and keenly aware of the possible adverse emotional response to this type of assessment because intraoral and extraoral areas are potentially personal and private areas of the body, especially in patients with a trauma history. Skin color can impact mucous membrane and gingiva color; therefore, to ensure cultural competence, the RDN should not make assumptions about a patient’s “normal” mucous membrane and gingiva color but should ask them what is “normal” for them.
Abdomen (Gastrointestinal)	The RDN should include open-ended questions that permit discussions of race-specific or family-specific gastrointestinal conditions or predispositions. The RDN should listen sensitively to patients’ concerns about their abdominal or gastrointestinal issues, even if the problem is not physiologically based. The RDN should be aware that functional gastrointestinal issues are common in patients with EDs, and some patients with functional gastrointestinal disorders may have a history of sexual trauma.

ED—eating disorder, RDN—registered dietitian nutritionist, TGNC—transgender and gender non-conforming, * Feedback was extracted and paraphrased (not verbatim).

**Table 7:** Round Two domains’ and components’ clinical relevance, consensus determination.

Domains and Components of Examination	Clinical Relevance Group Mean (N = 18)	Standard Deviation	Frequency (%) Rating 3 to 5	Consensus
**Domain 1 Anthropometrics**				
**Components A1, A5 Anthropometrics**				
A1 BMI	2.78	1.57	55.5	no
A5 Measuring weight when weight restoration is not a goal	2.83	1.04	55.6	no
**Domain 3 Vital Signs**				
**Component V6 Vital Signs**				
V6 New Component: Assessing capillary refill rate	3.67	1.14	88.9	yes
**Domain 4 Bone Loss and Injury, Body Fat, and Muscle Stores**				
**Component B6 Vital Signs**				
B6 New Component: Measuring grip strength	3.50	0.99	100.0	yes

BMI—body mass index.

## Data Availability

The data supporting the conclusions of this article will be made available by the corresponding author on request due to privacy reasons.

## Supplementary Materials

The following supporting information can be downloaded at: https://www.mdpi.com/article/10.3390/nu17091449/s1, the “Eating Disorders Nutrition Assessment Tool” Survey.

## Abbreviations

The following abbreviations are used in this manuscript:

ED	Eating Disorder
NFPE	Nutrition Focused Physical Examination
RDN	Registered Dietitian Nutritionist
IAEDP	International Association of Eating Disorder Professionals
LGBTQ+	Lesbian, Gay, Bisexual, Transgender, Queer, and Others
APA	American Psychiatric Association
BMI	Body Mass Index
SAMHSA	Substance Abuse and Mental Health Services Administration
TGD	Transgender and Gender Diverse
AED	Academy for Eating Disorders
AND	Academy of Nutrition and Dietetics
EDRD Pro	Eating Disorder Registered Dietitians and Professionals
IFEDD	International Federation of Eating Disorder Dietitians
CREDES	Conducting and Reporting of Delphi Studies
SD	Standard Deviation
PHP	Partial Hospitalization Program
IOP	Intensive Outpatient Program
BIPOC	Black, Indigenous, and People of Color
BPD	Bronchopulmonary Dysplasia
