# Supplementary material for: Development of a Trauma-Informed, Culturally Sensitive Eating-Disorder-Specific Nutrition-Focused Physical Examination Tool: A Modified Delphi Study
Source: Nutrients. 2025 Apr 25;17(9):1449. doi: 10.3390/nu17091449 (PMC12073526; doi:10.3390/nu17091449)
Supplement: Supplementary file 1 [file nutrients-17-01449-s001.zip › nutrients-3573738-supplementary.pdf]

# Eating Disorders Specific Assessment Tool Survey

Start of Block: Study Background and Instructions

Q1 **TITLE OF STUDY: DEVELOPMENT OF A TRAUMA-INFORMED, CULTURALLY SENSITIVE EATING DISORDERS-SPECIFIC NUTRITION ASSESSMENT TOOL: A DELPHI STUDY** **Study Background and Instructions** Thank you for your interest in participating in this study. The first aim of this study was to identify evidence-informed components of an eating disorders-specific nutrition assessment tool from previously published studies and resources. The items we are asking you to review and provide feedback on were identified from our literature search. Each evidence-informed component was organized by domain. The following survey questions address the study's second aim, which is to ask for your professional feedback on the **nine assessment domains, identified components, and approach for each domain**. The goal of this aim is to establish consensus among nutrition professionals who treat individuals with eating disorders on what should be included in an eating disorders-specific nutrition assessment tool. This prospective tool would complement a food and nutrition history, client history, laboratory assessment, and medical and diagnostic tests and provide a **trauma-informed, culturally sensitive alternative** to the traditional nutrition focused physical examination. It is not expected that you currently use all proposed assessment components (or any) in your practice, but instead, we are asking for your expert opinion regarding the **clinical** relevance of each item, and feedback on an assessment approach that could be performed in a **trauma-informed culturally sensitive manner** for all individuals, but specifically those with a potential trauma history and for diversity in age, sex, gender, sexual orientation, weight, size, religion, race, ethnicity, socioeconomic status, co-occurring mental illness, and physical and/or intellectual ability. The study will contain three rounds. The third round will be eliminated if consensus is reached after two rounds. In Round One, expert panelists will provide demographic information and respond to nine assessment domains' survey statements. Round One will also include open-ended questions requesting panelist feedback for assessment domain and component addition, omission, or modification. Lastly, Round One will include open-ended statements where panelists may provide feedback on any particular practice tools or techniques they may currently employ or would recommend to promote a trauma-informed, culturally specific assessment. Panelists do not have to respond to open-ended statements. Panelists will have two weeks to complete Round One and electronically submit their responses. An email reminder will be sent at the end of the first week. After Round One data collection, panelists will be provided with a summary of their previous responses and all other panelists' **deidentified** responses to permit panelists to compare their responses to other panelists' responses and revise them if desired. Panelists will be given an additional week to review their responses and revise them if desired. The round will then close and final data analysis will be conducted to determine if consensus was achieved on any

domains and components. Open-ended qualitative answers will be reviewed for potential additions, exclusions, modifications, and approach. Survey items from Round One that were added or revised by panelists and feedback on the assessment approach will be verified through additional literature review. These items will be added, omitted, or adjusted based on the results of a review of evidence and will be distributed with statements that did not reach consensus in Round One and revised statements based on panelist feedback and a review of evidence.    Rounds Two and Three will follow the same sequence as Round One (excluding demographic information). Items from Round One that reached consensus will be visible to panelists in Round Two but will be closed for further comments. Panelists will have two weeks to complete Round Two, with an email reminder sent at the end of the first week. In Round Two, data will be collected and analyzed. The process will be repeated for an additional round, Round Three if consensus has not been reached after Round Two. Results from Round Three will determine a final consensus of assessment domains, components, and approaches, and final revisions will be made and incorporated into the development of the ED-specific assessment tool.
The survey will take approximately 30 - 45 minutes to complete. You do not have to complete it at once. Your answers will automatically be saved. You will have two weeks to complete the survey once it has been emailed to you.

If you answer a question and choose to change it later, you can do that at any time prior to submitting your final responses by pressing the **back** button**.**

End of Block: Study Background and Instructions

---

Start of Block: Consent to Participate

Q2 **TITLE OF STUDY: DEVELOPMENT OF A TRAUMA-INFORMED, CULTURALLY SENSITIVE EATING DISORDERS-SPECIFIC NUTRITION ASSESSMENT TOOL: A DELPHI STUDY**    **Informed Consent**    The following is part of an informed consent process for a research study. It will provide information that will help you decide whether you want to participate in the study. It is your choice to take part or not. You may ask questions if there is anything in the form that is not clear to you. If you decide to participate, instructions at the end of the document will tell you what to do next. Your alternative to taking part in the research is not to take part in it.    **Who is conducting this research study and what is it about?**  You are being asked to take part in research conducted by Dr. Jane Ziegler. She is a Professor and Interim Chairperson of the Department of Clinical and Preventive Nutrition Sciences in the School of Health Professions at Rutgers University. The purpose of this study is to identify domains and components of a trauma-informed, culturally sensitive eating disorders-specific nutrition assessment tool and develop a consensus on the clinical relevance and approach to the assessment domains and components. We anticipate 20 subjects to take part in the research. **What will I be asked to do if I take part?**  This is a three-round e-Delphi study. The e-Delphi technique is used to build consensus among experts when there is limited empirical evidence available. Your role will be to review evidence-informed eating disorders assessment domains and components and complete a survey in three rounds (three surveys total). Each survey will

take approximately 30-45 minutes to complete. **What are the risks and/or discomforts I might experience if I take part in the study?** This study poses minimal potential risks to its participants. There are no consequences for choosing not to participate in the survey. Breach of confidentiality is a risk of harm, but a data security plan is in place to minimize such a risk. You may withdraw from the study at any time. If you decide to quit before you have finished the survey or round of the study, your answers will NOT be recorded. **Are there any benefits to me if I choose to take part in this study?** You will not receive any direct benefits for taking part in this research. You will be contributing to knowledge about using the physical assessment components as part of a complete nutrition assessment of individuals with eating disorders. The finalized results will be shared with you and other eating disorder treatment providers, which may benefit you in the future. **Will I be paid to take part in this study?** If you complete all three rounds of the survey, you will be able to enter a raffle to receive a $50.00 Amazon gift card. You will also have the opportunity to provide permission for the researcher to acknowledge your participation in the expert panel for written and oral presentations of this study. **How will information about me be kept private or confidential?** All efforts will be made to keep your responses confidential, but total confidentiality cannot be guaranteed. We will use a web-based survey tool to collect responses. In each round, you will be provided with an individualized report of your responses and a deidentified summary of responses from other study participants. We will download your responses to a secure file that requires a password to access. Only study staff will have access to the password. There is no plan to delete the responses. We plan to use the study data for some time. No information that can identify you will appear in any professional presentation or publication other than as an acknowledgment of participation, should you choose to disclose your identification for this purpose. **What will happen to the information I provide in the research after the study is over?** Deidentified responses may be used or distributed to investigators for other research without obtaining additional informed consent from you. **What will happen if I do not want to take part or decide later not to stay in the study?** Your participation is voluntary. If you choose to take part now, you may change your mind and withdraw later. In addition, you can choose to skip questions that you do not wish to answer. Your responses will not be recorded if you do not click on the 'submit' button after completing the form. **Who can I call if I have questions?** If you have questions about taking part in this study, you can contact the Principal Investigator: Dr. Jane Ziegler, ziegleja@shp.rutgers.edu. If you have questions, concerns, problems, information, or input about the research or would like to know your rights as a research subject, you can contact the Rutgers IRB or the Rutgers Human Subjects Protection Program via phone at (973) 972-3608 or (732) 235-2866 or (732) 235-9806 OR via email irboffice@research.rutgers.edu, or you can write us at 335 George Street, Liberty Plaza Suite 3200, New Brunswick, NJ 08901. Please print out or save this consent form if you would like a copy of it for your files. If you do not wish to take part in the research, delete this email. If you wish to take part in the study, follow the directions below: I acknowledge that I am 18 years of age or older and have read and understand the information by beginning this research. I agree to participate in the study,

knowing that I am free to withdraw my participation without penalty.    Select "Yes, I agree" button to confirm your agreement to take part in the research.

- ◯ Yes (4)
- ◯ No (5)

End of Block: Consent to Participate

---

Start of Block: Participant Eligibility

Q3 **Participant Eligibility**    To be eligible to participate in the study, you must have at least five years of experience treating individuals with eating disorders **OR** an advanced practice credential from the International Association of Eating Disorders Professionals (CEDS or CEDS-C). If you meet either of these criteria, please begin the survey. If you do not meet these criteria, please exit the survey. Thank you for your dedication to the treatment of individuals with eating disorders.

End of Block: Participant Eligibility

---

Start of Block: Name and Credentials

Q4 Please list your first and last name, your credentials, and contact email:

- ◯ First Name (1) ________________________________________________
- ◯ Last Name (2) ________________________________________________
- ◯ Professional Credentials (3) ________________________________________________
- ◯ Email (4) ________________________________________________

End of Block: Name and Credentials

---

Start of Block: Demographic Questions (Round One)

Q5 How many years have you worked in eating disorders treatment (the majority of your work is with individuals with eating disorders)?

○ Less than 5 years (1)

○ 5-9 years (2)

○ 10-14 years (3)

○ 15 or more years (4)

---

Q6 Which statement best describes your professional practice in **eating disorders** over the past five years?

○ Full-Time position (30 or more hours per week) (1)

○ Part-Time position (29 or less hours per week) (2)

○ Other (please describe) (6) ________________________________________________

---

Q7 Which statement best describes the treatment setting in which you treat individuals with eating disorders?

○ Specialized Eating Disorder Hospital Program (1)

○ Residential Facility (2)

○ Partial Hospitalization or Intensive Outpatient Program (3)

○ Outpatient Clinic or Private Practice (primarily eating disorders based) (4)

○ Other (please describe) (5) ________________________________________________

---

Q8 Which statement best describes your profession?

○ Registered Dietitian Nutritionist (5)

○ Other Nutrition Professional (please describe) (6)
________________________________________________

---

Q9 Do you have an advanced practice credential from the International Association of Eating Disorders Professionals?

○ Yes (1)

○ No (2)

*Skip To: Q11 If Do you have an advanced practice credential from the International Association of Eating Disorder... = No*

---

Q10 What is your advanced practice credential from the International Association of Eating Disorders Professionals?

○ CEDS (4)

○ CEDS-C (6)

---

Q11 If you have additional advanced practice credential(s), please list below.

________________________________________________________________

---

Q12 What is your gender?

- ◯ Male (1)
- ◯ Female (2)
- ◯ Transgender Male (4)
- ◯ Transgender Female (5)
- ◯ Non-Binary (6)
- ◯ Other (7)
- ◯ Prefer not to answer (3)

---

Q13 What is your age?

- ◯ Under 25 years old (1)
- ◯ 25-29 years old (2)
- ◯ 30-39 years old (3)
- ◯ 40-49 years old (4)
- ◯ 50-59 years old (5)
- ◯ Over 60 years old (6)
- ◯ Prefer not to answer (7)

**End of Block: Demographic Questions (Round One)**

---

**Start of Block: DOMAIN 1. ANTHROPOMETRICS**

Q14 **DOMAIN 1: Anthropometrics** The anthropometrics assessment domain includes measurements of body mass index (BMI), weight, height, and growth trajectory (in children and adolescents), and discussion of weight changes, body image, and weight-related "experiences" such as diets and weight stigma. **When weight restoration is a goal in eating disorders treatment,** anthropometric measurements are often used to evaluate medical status and

refeeding goals. **When weight restoration is not a goal in eating disorders treatment,** there are additional methods of addressing weight, shape, size, and weight-related experiences. Anthropometrics can be used to help detect malnutrition, excessive exercise, binge eating, weight-related eating disorder antecedents, current weight-related challenges, and non-nutrition-related weight-related circumstances.

---

Q15 Please rate the overall clinical relevance of DOMAIN 1 Anthropometrics in the nutrition assessment in eating disorders dietetics practice.

| | Strongly disagree (1) | Disagree (2) | Neither agree nor disagree (3) | Agree (4) | Strongly agree (5) |
|---|---|---|---|---|---|
| Domain 1 Anthropometrics is clinically relevant (1) | ○ | ○ | ○ | ○ | ○ |

---

Q16 Please rate the clinical relevance of the following DOMAIN 1 Anthropometrics components in the nutrition assessment in eating disorders dietetics practice.

| | Strongly disagree (1) | Disagree (2) | Neither agree nor disagree (3) | Agree (4) | Strongly agree (5) |
|---|---|---|---|---|---|
| Calculating BMI is clinically relevant (11) | ○ | ○ | ○ | ○ | ○ |
| Measuring height and growth trajectory in children and adolescents is clinically relevant (9) | ○ | ○ | ○ | ○ | ○ |
| Measuring weight when weight restoration is a treatment goal is clinically relevant (5) | ○ | ○ | ○ | ○ | ○ |
| Discussing weight changes, body image, and weight-related experiences when weight restoration is a treatment goal is clinically relevant (7) | ○ | ○ | ○ | ○ | ○ |
| Measuring weight when weight restoration is NOT a treatment goal is clinically relevant (6) | ○ | ○ | ○ | ○ | ○ |

| | | | | | |
|---|---|---|---|---|---|
| Discussing weight changes, body image, and weight-related experiences when weight restoration is NOT a treatment goal is clinically relevant (8) | ○ | ○ | ○ | ○ | ○ |

Q17 What modifications, additions, or exclusions would you make to Domain 1 Anthropometrics? Leave blank if you prefer not to answer or have no additional feedback.

________________________________________________________________

Q18 If you feel the anthropometrics assessment domain is clinically relevant, please provide feedback on any particular practice tools or techniques you may currently employ or would recommend to promote a trauma-informed, culturally specific assessment. Leave blank if you prefer not to answer or have no additional feedback.

________________________________________________________________

________________________________________________________________

________________________________________________________________

________________________________________________________________

________________________________________________________________

**End of Block: DOMAIN 1. ANTHROPOMETRICS**

**Start of Block: DOMAIN 2. GENERAL SURVEY, COGNITION, NEUROPSYCHIATRIC SYMPTOMS**

Q19 **DOMAIN 2: General Survey, Cognition, and Neuropsychiatric Symptoms** The general survey, cognition, and neuropsychiatric symptoms domain includes an assessment through discussion and observation with the client of the level of consciousness, alertness, answers to questions, energy level, thinking, understanding, memory, mood, affect, sleep, and other physiological brain-based signs and symptoms of eating disorder behaviors. The **general survey** is used by dietitian nutritionists for clients with eating disorders to assess for reduced general level of consciousness, engagement and alertness, lack of age-appropriate behavior and answers to questions, and general feelings of weakness and fatigue. Symptoms in this domain may result from macronutrient and micronutrient deficiencies, excesses, and imbalances, inadequate calorie intake, dehydration, excessive exercise, and night eating. The **cognitive assessment** evaluates for diminished thinking and understanding, dizziness, slowed speech, and memory loss. These symptoms may result from macronutrient and micronutrient deficiencies, excesses, and imbalances, inadequate calorie intake, dehydration, excessive exercise, and night eating. **Neuropsychiatric symptoms** may include depressed mood, anxiety, flat affect, apathy, agitation, dissociation, migraines, seizures, and sleep disorders. These symptoms may result from possible thiamine, pyridoxine, vitamin B12, iron deficiencies, macronutrient deficiencies, excesses, or imbalances, inadequate calorie intake, dehydration, thyroid medication misuse, hypothyroidism, excessive exercise, and night eating. This assessment domain also includes attention to potential medication effects and side effects, non-nutritional issues, pre-existing psychiatric conditions, trauma, and neurodivergence including autism spectrum disorders, attention deficit disorders, and others.

---

Q20 Please rate the overall clinical relevance of DOMAIN 2 General Survey, Cognition, and Neuropsychiatric Symptoms in the nutrition assessment in eating disorders dietetics practice.

| | Strongly disagree (1) | Disagree (2) | Neither agree nor disagree (3) | Agree (4) | Strongly agree (5) |
|---|---|---|---|---|---|
| Domain 2 General Survey, Cognition, and Neuropsychiatric Symptoms is clinically relevant (1) | ○ | ○ | ○ | ○ | ○ |

---

Q21 Please rate the clinical relevance of the following DOMAIN 2 General Survey, Cognition, and Neuropsychiatric Symptoms components in the nutrition assessment in eating disorders dietetics practice.

| | Strongly disagree (1) | Disagree (2) | Neither agree nor disagree (3) | Agree (4) | Strongly agree (5) |
|---|---|---|---|---|---|
| A general survey assessment is clinically relevant (1) | ○ | ○ | ○ | ○ | ○ |
| A cognitive assessment is clinically relevant (6) | ○ | ○ | ○ | ○ | ○ |
| An assessment of neuropsychiatric symptoms is clinically relevant (7) | ○ | ○ | ○ | ○ | ○ |

Q22 What modifications, additions, or exclusions would you make to Domain 2 General Survey, Cognition, and Neuropsychiatric Symptoms? Leave blank if you prefer not to answer or have no additional feedback.

________________________________________________________________

Q23 If you feel the general survey, cognition, and neuropsychiatric symptoms assessment domain is clinically relevant, please provide feedback on any particular practice tools or techniques you may currently employ or would recommend to promote a trauma-informed, culturally specific assessment. Leave blank if you prefer not to answer or have no additional feedback.

________________________________________________________________

**End of Block: DOMAIN 2. GENERAL SURVEY, COGNITION, NEUROPSYCHIATRIC SYMPTOMS**

Start of Block: DOMAIN 3. VITAL SIGNS

Q24 **DOMAIN 3: Vital Signs** The vital signs assessment domain includes a discussion with the client and measurements of blood pressure, orthostatic blood pressure, heart rate (radial pulse), and temperature (with explicit patient or guardian permission). Signs and symptoms such as hypothermia, cool extremities, orthostatic hypotension, hypertension, bradycardia, tachycardia, lightheadedness, chest pain, fatigue, and hot flashes may result from malnutrition, weight loss, purging, excessive exercise, laxative or diuretic abuse, caffeine abuse, supplement misuse, drug use, thyroid medication misuse, overhydration, electrolyte imbalances (hypokalemia, hypomagnesemia, hypophosphatemia), refeeding syndrome, hypothyroidism (euthyroid sick syndrome), hypometabolism, hypermetabolism, and iron deficiency.

---

Q25 Please rate the overall clinical relevance of DOMAIN 3 Vital Signs in the nutrition assessment in eating disorders dietetics practice.

| | Strongly disagree (1) | Disagree (2) | Neither agree nor disagree (3) | Agree (4) | Strongly agree (5) |
|---|---|---|---|---|---|
| Domain 3 Vital Signs is clinically relevant (1) | ○ | ○ | ○ | ○ | ○ |

---

Q26 Please rate the clinical relevance of the following Domain 3 Vital Signs components in the nutrition assessment in eating disorders dietetics practice.

| | Strongly disagree (1) (2) | Somewhat disagree (2) (3) | Neither agree nor disagree (3) (4) | Somewhat agree (4) (5) | Strongly agree (5) (6) |
|---|---|---|---|---|---|
| Discussion of vital signs and symptoms is clinically relevant (5) | ○ | ○ | ○ | ○ | ○ |
| Measuring blood pressure is clinically relevant (1) | ○ | ○ | ○ | ○ | ○ |
| Measuring orthostatic blood pressure is clinically relevant (4) | ○ | ○ | ○ | ○ | ○ |
| Measuring heart rate using radial pulse is clinically relevant (2) | ○ | ○ | ○ | ○ | ○ |
| Measuring temperature is clinically relevant (3) | ○ | ○ | ○ | ○ | ○ |

Q27 What modifications, additions, or exclusions would you make to Domain 3 Vital Signs? Leave blank if you prefer not to answer or have no additional feedback.

________________________________________________________________

________________________________________________________________

________________________________________________________________

________________________________________________________________

________________________________________________________________

Q28 If you feel the vital signs assessment domain is clinically relevant, please provide feedback on any particular practice tools or techniques you may currently employ or would recommend to promote a trauma-informed, culturally specific assessment. Leave blank if you prefer not to answer or have no additional feedback.

________________________________________________________________

________________________________________________________________

________________________________________________________________

________________________________________________________________

________________________________________________________________

**End of Block: DOMAIN 3. VITAL SIGNS**

**Start of Block: DOMAIN 4. BONE LOSS AND INJURY, BODY FAT AND MUSCLE STORES**

Q29 **DOMAIN 4: Bone Loss and Injury, Body Fat and Muscle Stores** The bone loss and injury, fat and muscle stores domain includes an assessment of bone health, fat, and muscle stores. **Bone health assessment** includes a discussion of previous or current injuries such as stress fractures or other fractures, other bone-related injuries, bone pain, poor bone growth, bone weakness (osteomalacia), and bone density conditions such as osteopenia and osteoporosis diagnosed through bone density (DEXA) scan. Bone injury, weakness, pain, loss, and poor growth can result from macronutrient and micronutrient deficiencies or imbalances, calorie insufficiency, weight loss, purging, laxative abuse, excessive exercise, and diminished hormone levels. This assessment domain also includes attention to potential skeletal side effects from medications and non-nutritional or age-related bone loss in older, post-menopausal women. Body fat stores and distribution can change throughout the development and course of an eating disorder, and during the recovery process (including refeeding when necessary). Body fat assessment may include a discussion of changes in body fat stores. **Body fat assessment** may also include observation (with explicit patient or guardian permission) of fat stores in the orbital region. Due to the sensitivity of the topic of body fat, discussion may need to be limited. Body fat loss may result from macronutrient and calorie restriction, weight loss, excessive exercise, purging, laxative and diuretic abuse, drug use, insulin misuse, supplement misuse, and thyroid medication misuse. Body fat gain and redistribution may occur during refeeding, and from restrictive eating, binge eating other non-hunger-based eating, and excessive exercise. **Body muscle assessment** includes a discussion of changes in muscle

stores, muscle pain, weakness, inflammation, cramps, rhabdomyolysis, peripheral neuropathy (sensations of pins and needles), and joint pain. Muscle assessment may also include observation (with explicit patient or guardian permission) of muscle loss in interosseous hand muscles and in the temple region. Muscle loss may result from macronutrient and calorie restriction, weight loss, excessive exercise, purging, laxative and diuretic abuse, and supplement misuse. Muscle gain may result from macronutrient imbalances, supplement misuse, and excessive (weight lifting) exercise.     Fat and muscle amount and distribution may also be genetic, a medication side effect, a result of various types of hormone replacement therapy, or from other non-nutritional influences.

---

Q30 Please rate the overall clinical relevance of DOMAIN 4 Bone Loss and Injury, Body Fat and Muscle Stores in the nutrition assessment in eating disorders dietetics practice.

| | Strongly disagree (1) | Disagree (2) | Neither agree nor disagree (3) | Agree (4) | Strongly agree (5) |
|---|---|---|---|---|---|
| Domain 4 Bone Loss and Injury, Body Fat and Muscle Stores is clinically relevant (1) | o | o | o | o | o |

---

Q31 Please rate the clinical relevance of the following Domain 4 Bone Loss and Injury, Body Fat, and Muscle Stores components in the nutrition assessment in eating disorders dietetics practice.

| | Strongly Disagree (1) | Disagree (2) | Neither Agree nor Disagree (3) | Agree (4) | Strongly Agree (5) |
|---|---|---|---|---|---|
| Discussion of bone health is clinically relevant (1) | ○ | ○ | ○ | ○ | ○ |
| Discussion of body fat changes is clinically relevant (2) | ○ | ○ | ○ | ○ | ○ |
| Observation of body fat changes is clinically relevant (4) | ○ | ○ | ○ | ○ | ○ |
| Discussion of body muscle changes and symptoms is clinically relevant (3) | ○ | ○ | ○ | ○ | ○ |
| Observation of body muscle changes and symptoms is clinically relevant (5) | ○ | ○ | ○ | ○ | ○ |

Q32 What modifications, additions, or exclusions  would you make to Domain 4 Bone Loss and Injury, Body Fat and Muscle Stores? Leave blank if you prefer not to answer or if you have no additional feedback.

________________________________________________________________

Q33 If you feel the bone loss and injury, body fat, and muscle stores assessment domain is clinically relevant, please provide feedback on any particular practice tools or techniques you may currently employ or would recommend to promote a trauma-informed, culturally specific assessment. Leave blank if you prefer not to answer or have no additional feedback.

________________________________________________________________

End of Block: DOMAIN 4. BONE LOSS AND INJURY, BODY FAT AND MUSCLE STORES

---

Start of Block: DOMAIN 5. HYDRATION STATUS

Q34 **DOMAIN 5: Hydration Status** The hydration status domain includes an assessment of dehydration, overhydration, and hydration shifts through discussion of types and amounts of fluid intake, and eating disorder behaviors. It also includes an assessment of refeeding goals as refeeding syndrome can cause severe electrolyte imbalances. Adequate hydration results in normal mucous membranes, normal skin turgor, and clear pale yellow urine. This domain includes a discussion and observation (with explicit patient or guardian permission) of potential signs and symptoms of **dehydration** such as sunken eyes, hyperpigmentation under the eyes, loss of skin turgor, dry, cracked lips, extreme thirst, headache, fatigue, dizziness, confusion, weight loss, concentrated urine, and infrequent urination. These signs and symptoms can result from fluid restriction, laxative and diuretic abuse, purging, and excessive exercise. This domain also includes a discussion and observation (with explicit patient or guardian permission) of potential signs and symptoms of **overhydration** such as puffy eyes, nausea, vomiting, headache, confusion, disorientation, muscle cramps, distended abdomen, weight gain, pale colorless urine, and frequent urination. These signs and symptoms can result from water loading, or cessation of purging, laxative, or diuretic abuse. Lastly, it includes a discussion and observation (with explicit patient or guardian permission) of potential signs and symptoms of **abdominal, and peripheral edema** in the extremities - hands, feet, and ankles. Edema, fluid buildup in the tissues causing swelling, can result from low albumin levels, protein or thiamine deficiency, electrolyte imbalances, cessation of purging, laxative or diuretic abuse, bingeing, and refeeding (including refeeding syndrome). Dehydration, overhydration, and edema may also result from medications and non-nutritional causes.

Q35 Please rate the overall clinical relevance of DOMAIN 5 Hydration Status in the nutrition assessment in eating disorders dietetics practice.

| | Strongly disagree (1) | Disagree (2) | Neither agree nor disagree (3) | Agree (4) | Strongly agree (5) |
|---|---|---|---|---|---|
| Domain 5 Hydration Status is clinically relevant (1) | ○ | ○ | ○ | ○ | ○ |

Q36 Please rate the clinical relevance of the following Domain 5 Hydration Status components in the nutrition assessment in eating disorders dietetics practice.

| | Strongly disagree (1) | Disagree (2) | Neither agree nor disagree (3) | Agree (4) | Strongly agree (5) |
|---|---|---|---|---|---|
| Discussion of dehydration signs and symptoms is clinically relevant (1) | ○ | ○ | ○ | ○ | ○ |
| Observation of dehydration signs and symptoms is clinically relevant (6) | ○ | ○ | ○ | ○ | ○ |
| Discussion of overhydration signs and symptoms is clinically relevant (2) | ○ | ○ | ○ | ○ | ○ |
| Observation of overhydration signs and symptoms is clinically relevant (7) | ○ | ○ | ○ | ○ | ○ |
| Discussion of signs and symptoms of abdominal edema is clinically relevant (3) | ○ | ○ | ○ | ○ | ○ |
| Observation of signs and symptoms of abdominal edema is clinically relevant (5) | ○ | ○ | ○ | ○ | ○ |

| | | | | | |
|---|---|---|---|---|---|
| Discussion of signs and symptoms of peripheral edema is clinically relevant (4) | ○ | ○ | ○ | ○ | ○ |
| Observation of signs and symptoms of peripheral edema is clinically relevant (8) | ○ | ○ | ○ | ○ | ○ |

Q37 What modifications, additions, or exclusions would you make to Domain 5 Hydration Status? Leave blank if you prefer not to answer or have no additional feedback.

________________________________________________________________

Q38 If you feel the hydration status assessment domain is clinically relevant, please provide feedback on any particular practice tools or techniques you may currently employ or would recommend to promote a trauma-informed, culturally specific assessment. Leave blank if you prefer not to answer or have no additional feedback.

________________________________________________________________

**End of Block: DOMAIN 5. HYDRATION STATUS**

**Start of Block: DOMAIN 6. SKIN, HANDS, AND NAILS**

Q39 **DOMAIN 6: Skin, Hands, and Nails** The skin, hands, and nails domain includes an assessment of skin, hands, and nail health. Normal skin should appear uniform in color, texture, moisture, and temperature. Normal hands should also appear uniform in color and have sufficient interosseous muscle with no bruising or lesions. Normal nails should be smooth and normal in color and shape, with less than two seconds of capillary refill time. The **skin and hands assessment** includes a discussion and observation (with explicit patient or guardian permission) of the skin (face, arms) and hands. Signs and symptoms may include poor wound healing, pallor, xerosis, acanthosis nigricans, hirsutism, dermatitis, acne, loss of turgor, cool

temperature, general bruising, bruising over bony prominences, striae distensae (stretch marks), petechiae, cyanosis, lesions, burns and cuts (signs of self-harm), carotenoderma on palms of hands, Russell's sign (scars or calluses on backs of hands or knuckles), and interosseous muscle loss.    These signs and symptoms may result from inadequate calorie intake, macronutrient deficiencies, excesses, or imbalances, dehydration, micronutrient deficiencies (iron, folate, vitamin B12, vitamin A, iron, and zinc), excessive intake of beta-carotene containing foods, dermatillomania (skin picking), weight gain or weight loss, purging, excessive exercise, binge eating, and self-harm.    The **nails assessment** includes a discussion and observation (with explicit patient or guardian permission) of the fingernails and cuticles. Ridges, koilonychia (spoon-shaped nails), dry, peeling, or short nails, pale nail bed, bleeding cuticles, and slow capillary refill time may result from inadequate calorie intake, protein deficiency, micronutrient deficiencies (iron, zinc, folate, magnesium, and selenium), nail and cuticle biting, and hypovolemia.    Other medical illnesses and non-nutrition-related circumstances can cause changes in skin and nails. Additionally, there are genetic, ethnic, and racial variations in skin and nails.

Q40 Please rate the overall clinical relevance of DOMAIN 6 Skin, Hands, and Nails in the nutrition assessment in eating disorders dietetics practice.

| | Strongly disagree (1) | Disagree (2) | Neither agree nor disagree (3) | Agree (4) | Strongly agree (5) |
|---|---|---|---|---|---|
| Domain 6 Skin, Hands, and Nails is clinically relevant (1) | ○ | ○ | ○ | ○ | ○ |

Q41 Please rate the clinical relevance of the following Domain 6 Skin, Hands, and Nails components in the nutrition assessment in eating disorders dietetics practice.

| | Strongly disagree (1) | Disagree (2) | Neither agree nor disagree (3) | Agree (4) | Strongly agree (5) |
|---|---|---|---|---|---|
| Discussion of changes in skin and hands is clinically relevant (1) | ○ | ○ | ○ | ○ | ○ |
| Observation of changes in skin and hands is clinically relevant (2) | ○ | ○ | ○ | ○ | ○ |
| Discussion of changes in nails is clinically relevant (3) | ○ | ○ | ○ | ○ | ○ |
| Observation of changes in nails is clinically relevant (4) | ○ | ○ | ○ | ○ | ○ |

Q42 What modifications, additions, or exclusions would you make to Domain 6 Skin, Hands, and Nails? Leave blank if you prefer not to answer or have no additional feedback.

________________________________________________________________

Q43 If you feel the skin, hands, and nails assessment domain is clinically relevant, please provide feedback on any particular practice tools or techniques you may currently employ or would recommend to promote a trauma-informed, culturally specific assessment. Leave blank if you prefer not to answer or have no additional feedback.

________________________________________________________________

End of Block: DOMAIN 6. SKIN, HANDS, AND NAILS

Start of Block: DOMAIN 7. HAIR, EYELASHES, EYEBROWS, AND EYES

Q44 **DOMAIN 7: Hair, Eyelashes, Eyebrows, and Eyes** An assessment of the hair, eyelashes, eyebrows, and eyes can detect signs and symptoms of micronutrient and macronutrient deficiencies, calorie insufficiency, trichotillomania, and endocrine disorders. Normal hair, eyelashes, and eyebrows are uniform in color, texture, and amount. Healthy eyes have sufficient moisture, white sclera, light pink conjunctiva, normal skin turgor and fat pads surrounding the eyes, and no nutrition-related vision impairment. The **hair, eyelashes, and eyebrows assessment** includes a discussion and observation (with explicit patient or guardian permission) of hair on the head and torso, eyelashes, and eyebrows. Alopecia, easily pluckable, brittle, dry hair, loss of eyebrows, eyelashes, and lanugo on face and torso may result from protein, iron, zinc, and essential fatty acid deficiency, hypothyroidism, severe malnutrition, and trichotillomania (hair pulling). The **eyes assessment** includes a discussion and observation (with explicit patient or guardian permission) of vision, sclera, conjunctiva color and moisture, skin turgor around the eyes, and fat pads. Diminished vision quality, dry or pale conjunctiva, skin turgor loss around the eyes, subconjunctival hemorrhage, orbital fat atrophy leading to lagophthalmos (inability to completely close eyes) may result from micronutrient deficiencies (thiamine, folate, vitamin B12, vitamin A, and iron), dehydration, body fat loss, and purging. Changes in facial and body hair amount and distribution may also result from non-nutritional influences including hormonal issues and hormone replacement therapy in gender-affirming treatment.

---

Q45 Please rate the overall clinical relevance of DOMAIN 7 Hair, Eyelashes, Eyebrows, and Eyes in the nutrition assessment in eating disorders dietetics practice.

| | Strongly disagree (1) | Disagree (2) | Neither agree nor disagree (3) | Agree (4) | Strongly agree (5) |
|---|---|---|---|---|---|
| Domain 7 Hair, Eyelashes, Eyebrows, and Eyes is clinically relevant (1) | ○ | ○ | ○ | ○ | ○ |

---

Q46 Please rate the clinical relevance of the following Domain 7 Hair, Eyelashes, Eyebrows, and Eyes components in the nutrition assessment in eating disorders dietetics practice.

| | Strongly disagree (1) | Disagree (2) | Neither agree nor disagree (3) | Agree (4) | Strongly agree (5) |
|---|---|---|---|---|---|
| Discussion of changes in hair is clinically relevant (1) | ○ | ○ | ○ | ○ | ○ |
| Observation of changes in hair is clinically relevant (4) | ○ | ○ | ○ | ○ | ○ |
| Discussion of changes in eyelashes and eyebrows is clinically relevant (2) | ○ | ○ | ○ | ○ | ○ |
| Observation of changes in eyelashes and eyebrows is clinically relevant (5) | ○ | ○ | ○ | ○ | ○ |
| Discussion of changes in eyes is clinically relevant (3) | ○ | ○ | ○ | ○ | ○ |
| Observation of changes in eyes is clinically relevant (6) | ○ | ○ | ○ | ○ | ○ |

Q47 What modifications, additions, or exclusions would you make to Domain 7 Hair, Eyelashes, Eyebrows, and Eyes? Leave blank if you prefer not to answer.

________________________________________________________________

Q48 If you feel the hair, eyelashes, eyebrows, and eyes assessment domain is clinically relevant, please provide feedback on any particular practice tools or techniques you may currently employ or would recommend to promote a trauma-informed, culturally specific assessment. Leave blank if you prefer not to answer.

________________________________________________________________

**End of Block: DOMAIN 7. HAIR, EYELASHES, EYEBROWS, AND EYES**

---

**Start of Block: DOMAIN 8. INTRAORAL, EXTRAORAL, AND NECK**

Q49 **DOMAIN 8: Intraoral, Extraoral, and Neck** The intraoral, extraoral, and neck domain includes an assessment of the mouth and neck regions. A healthy mouth has a normal amount of healthy teeth, pink smooth lips with no sores, pink gums, red, moist tongue with papillae, and a rough appearance. A healthy neck is smooth with symmetry. Healthy parotid glands are unilobular with a triangular shape, and no protrusion. The **intraoral assessment** includes a discussion and observation (with explicit patient or guardian permission) of teeth, gums, mucosa, tongue, and breath. Intraoral signs and symptoms may include gingivitis, dental caries, tooth decay, tooth sensitivity, broken teeth, missing teeth, tooth enamel loss, mouth sores, swollen bleeding gums, palatal scratches, pallor, inflamed mucosa, depapillated tongue, dark red (magenta) tongue, mouth dryness, mouth burning sensation, halitosis, hyposalivation, oropharyngeal dysphasia, and increased or diminished gag reflex. These intraoral signs and symptoms can result from micronutrient deficiencies (iron, zinc, calcium, selenium, magnesium, copper, riboflavin, niacin, vitamin B6, vitamin B12, folate, vitamin C, vitamin D) ketoacidosis, excessive carbonated and caffeinated beverages, highly sweetened foods, chewing gum, dehydration, bingeing, purging, chewing and spitting, chewing hard foods (ice, hard candy) or non-food items, using hard implements to purge, brushing teeth after purging, and low muscle tone (from weight loss). The **extraoral assessment** includes a discussion and observation (with explicit patient or guardian permission) of the lips. Angular cheilitis, angular stomatitis, and external mouth sores can result from dehydration, bingeing, mechanical trauma from purging, purging of acidic stomach contents, chewing and spitting, and micronutrient deficiencies (iron, zinc, riboflavin, niacin, vitamin B6, vitamin B12, and folate) The oral assessment also includes a discussion of the sense of taste and voice quality. Hypogeusia (lack of taste) can result from zinc deficiency, taste papillae reduction, or purging. A weak or hoarse voice can result from malnutrition or purging. The **neck assessment** includes a discussion and observation (with explicit patient or guardian permission) of the neck. Parotid gland pain and enlargement can

result from bingeing, purging, chewing, and spitting.    Intraoral and extraoral signs and symptoms can also result from medications and non-nutritional causes.

Q50 Please rate the overall clinical relevance of DOMAIN 9 Intraoral, Extraoral, and Neck in the nutrition assessment in eating disorders dietetics practice.

| | Strongly disagree (1) | Disagree (2) | Neither agree nor disagree (3) | Agree (4) | Strongly agree (5) |
|---|---|---|---|---|---|
| Domain 8 Intraoral, Extraoral, and Neck is clinically relevant (1) | o | o | o | o | o |

Q51 Please rate the clinical relevance of the following Domain 8 Intraoral, Extraoral, and Neck components in the nutrition assessment in eating disorders dietetics practice.

| | Strongly disagree (1) | Disagree (2) | Neither agree nor disagree (3) | Agree (4) | Strongly agree (5) |
|---|---|---|---|---|---|
| Discussion of intraoral changes is clinically relevant (1) | ○ | ○ | ○ | ○ | ○ |
| Observation of intraoral changes is clinically relevant (2) | ○ | ○ | ○ | ○ | ○ |
| Discussion of extraoral changes is clinically relevant (3) | ○ | ○ | ○ | ○ | ○ |
| Observation of extraoral changes is clinically relevant (4) | ○ | ○ | ○ | ○ | ○ |
| Discussion of the neck (parotid glands) is clinically relevant (5) | ○ | ○ | ○ | ○ | ○ |
| Observation of the neck (parotid glands) is clinically relevant (6) | ○ | ○ | ○ | ○ | ○ |

Q52 What modifications, additions, or exclusions would you make to Domain 8 Intraoral, Extraoral, and Neck? Leave blank if you prefer not to answer or have no additional feedback.

________________________________________________________________

Q53 If you feel the intraoral, extraoral, and neck assessment domain is clinically relevant, please provide feedback on any particular practice tools or techniques you may currently employ or would recommend to promote a trauma-informed, culturally specific assessment. Leave blank if you prefer not to answer or have no additional feedback.

________________________________________________________________

End of Block: DOMAIN 8. INTRAORAL, EXTRAORAL, AND NECK

Start of Block: DOMAIN 9. ABDOMEN

Q54 **DOMAIN 9: Abdomen (Gastrointestinal)** The abdomen (gastrointestinal) domain includes an assessment of pre-existing and co-occurring gastrointestinal illnesses, current gastrointestinal symptoms, and abdominal appearance. If symptoms are present, the abdominal assessment helps distinguish between functional, structural, and psychosomatic gastrointestinal issues and identifies signs and symptoms of eating disorder behaviors. A healthy abdomen is soft, has a flat, round, or scaphoid appearance, normal bowel sounds, and minimal self-reported discomfort. The **abdominal (gastrointestinal) assessment** includes a discussion of all gastrointestinal symptoms. Symptoms may include: Esophagus - esophagitis, esophageal dysmotility, gastroesophageal reflux disease, heartburn, acid regurgitation, epigastric tenderness, Mallory–Weiss tears, esophageal rupture Stomach - gastritis, acute gastric dilation, dyspepsia, superior mesenteric artery syndrome, delayed gastric emptying, nausea, early satiety, abdominal pain, stomach distension, fluid accumulation Small and large intestines - bloating, cramping, electrolyte imbalances, constipation, diarrhea, cathartic colon Rectum, anus - flatulence, rectal bleeding, hemorrhoids, rectal prolapse, anal fissure Other - pelvic floor dysfunction Gastrointestinal signs and symptoms can result from malnutrition, weight loss, weight gain, refeeding, overconsumption of foods high in fiber and fluids, laxative or diuretic abuse, cessation of laxatives or diuretics, bingeing, purging with acid exposure, injury from purging implements, overexercise, or pre-existing illness. The **abdominal (gastrointestinal) assessment** also includes a discussion and observation (with explicit patient or guardian permission) of the abdomen. An extreme scaphoid abdomen may result from weight loss or excessive exercise. Abdominal distension or firmness may result from weight gain, bingeing, food intolerance, constipation, or rebound edema.

Q55 Please rate the overall clinical relevance of DOMAIN 9 Abdomen (Gastrointestinal) in the nutrition assessment in eating disorders dietetics practice.

| | Strongly disagree (1) | Disagree (2) | Neither agree nor disagree (3) | Agree (4) | Strongly agree (5) |
|---|---|---|---|---|---|
| Domain 9 Abdomen (Gastrointestinal) is clinically relevant (1) | ○ | ○ | ○ | ○ | ○ |

Q56 Please rate the clinical relevance of the following Domain 9 Abdomen (Gastrointestinal) components in the nutrition assessment in eating disorders dietetics practice.

| | Strongly disagree (1) | Disagree (2) | Neither agree nor disagree (3) | Agree (4) | Strongly agree (5) |
|---|---|---|---|---|---|
| Discussion of gastrointestinal illness, signs and symptoms is clinically relevant (1) | ○ | ○ | ○ | ○ | ○ |
| Discussion of abdomen is clinically relevant (3) | ○ | ○ | ○ | ○ | ○ |
| Observation of abdomen is clinically relevant (2) | ○ | ○ | ○ | ○ | ○ |

Q57 What modifications, additions, or exclusions would you make to Domain 9 Abdomen (Gastrointestinal)? Leave blank if you prefer not to answer or have no additional feedback.

________________________________________________________________

Q58 If you feel the abdomen assessment (gastrointestinal) domain is clinically relevant, please provide feedback on any particular practice tools or techniques you may currently employ or would recommend to promote a trauma-informed, culturally specific assessment. Leave blank if you prefer not to answer or have no additional feedback.

________________________________________________________________

End of Block: DOMAIN 9. ABDOMEN

Start of Block: Block 14

Q59 If you would like to be included in a raffle for a $50.00 Amazon gift card for your participation, please add your email here.

________________________________________________________________

End of Block: Block 14
